# Genomic Resources for Darters (Percidae: Etheostominae) Provide Insight into Postzygotic Barriers Implicated in Speciation

**DOI:** 10.1093/molbev/msz260

**Published:** 2019-11-05

**Authors:** Rachel L Moran, Julian M Catchen, Rebecca C Fuller

**Affiliations:** 1 Program in Ecology, Evolution, and Conservation Biology, Department of Animal Biology, University of Illinois at Urbana-Champaign, Champaign, IL; 2 Department of Ecology, Evolution, and Behavior, University of Minnesota, St. Paul, MN

**Keywords:** speciation, hybridization, postzygotic isolation, genetic incompatibilities, chromosomal rearrangements

## Abstract

Comparative genomic approaches are increasingly being used to study the evolution of reproductive barriers in nonmodel species. Although numerous studies have examined prezygotic isolation in darters (Percidae), investigations into postzygotic barriers have remained rare due to long generation times and a lack of genomic resources. Orangethroat and rainbow darters naturally hybridize and provide a remarkable example of male-driven speciation via character displacement. Backcross hybrids suffer from high mortality, which appears to promote behavioral isolation in sympatry. To investigate the genomic architecture of postzygotic isolation, we used Illumina and PacBio sequencing to generate a chromosome-level, annotated assembly of the orangethroat darter genome and high-density linkage maps for orangethroat and rainbow darters. We also analyzed genome-wide RADseq data from wild-caught adults of both species and laboratory-generated backcrosses to identify genomic regions associated with hybrid incompatibles. Several putative chromosomal translocations and inversions were observed between orangethroat and rainbow darters, suggesting structural rearrangements may underlie postzygotic isolation. We also found evidence of selection against recombinant haplotypes and transmission ratio distortion in backcross hybrid genomes, providing further insight into the genomic architecture of genetic incompatibilities. Notably, regions with high levels of genetic divergence between species were enriched for genes associated with developmental and meiotic processes, providing strong candidates for postzygotic isolating barriers. These findings mark significant contributions to our understanding of the genetic basis of reproductive isolation between species undergoing character displacement. Furthermore, the genomic resources presented here will be instrumental for studying speciation in darters, the most diverse vertebrate group in North America.

## Introduction

Identifying reproductive isolating barriers that prevent gene exchange between taxa remains a central goal of speciation research (Coyne and Orr 2004; Butlin et al. 2012). Understanding the genetic basis of such barriers presents a particular challenge in nonmodel organisms that are not easily crossed in the laboratory (Orr and Presgraves 2000). However, second- and third-generation sequencing technology have made it possible to take a comparative genomic approach to identify barriers to gene flow, even in cases where traditional quantitative genetic approaches are not feasible (Butlin 2010; Wolf et al. 2010; Ellegren 2014). With these new technologies, we can greatly advance our understanding of the roles that epistasis, genomic structural variants, and recombination play in speciation and the maintenance of reproductive isolation in the face of gene flow.

Genetic incompatibilities underlying postzygotic barriers have been identified across a wide range of taxa (e.g., *Mimulus*: Martin and Willis 2010; *Arabidopsis*: Kradolfer et al. 2013; *Drosophila*: Coyne 1984; Moehring et al. 2006; grasshoppers: Virdee and Hewitt 1992; lake whitefish: Rogers and Bernatchez 2006; *Ficedula* flycatchers: Sætre et al. 2003; mice: Good et al. 2008). Two mechanisms that appear to be commonly implicated in the evolution of genetic incompatibilities and postzygotic isolation are chromosomal rearrangements and negative epistatic interactions. Chromosomal rearrangements include inversions, fusions, and translocations in a species relative to the ancestral state. If two lineages diverge in gene order collinearity (i.e., synteny) along homologous chromosomes due to such rearrangements, it can cause problems with chromosome pairing and crossing over during meiosis (thus suppressing recombination; Lai et al. 2005; McGaugh and Noor 2012). Hybrid offspring are more likely to be aneuploid in regions of the genome associated with rearrangements, which often results in negative fitness effects and sometimes in complete inviability (Noor et al. 2001; Rieseberg 2001). Negative epistatic interactions (e.g., Dobzhansky–Muller incompatibilities; Dobzhansky 1936; Muller 1942) can occur when new alleles arise in each of two diverging lineages and cause no negative impact on fitness within each lineage, but result in decreased fitness when brought together due to hybridization (Orr and Presgraves 2000; Turelli and Orr 2000; Coyne and Orr 2004).

Investigations into the mechanism of genetic incompatibilities and the genome-wide frequency and distribution of loci involved in postzygotic barriers between species have been conducted in a number of model species (sunflowers: Rieseberg et al. 1999; Gardner et al. 2000; mice: Teeter et al. 2010; threespine stickleback: Hohenlohe et al. 2012; swordtail fishes: Schumer et al. 2014; *Drosophila*: Pool 2015; *Populus*: Christe et al. 2016), but we lack data on the genetic basis of reproductive isolation in systems where character displacement drives speciation (Garner et al. 2018). Here, we generate the first genome and linkage maps for darters (Percidae: Etheostominae). We use these tools in conjunction with genome-wide sequence data from laboratory-generated backcross hybrids to investigate the genomic architecture of postzygotic isolation in two groups of darters that have become emerging models for the study of speciation via reproductive character displacement and agonistic character displacement (Zhou and Fuller 2014; Moran et al. 2017; Moran and Fuller 2018a, 2018b): the orangethroat darter (*Etheostoma spectabile*) and the rainbow darter (*Etheostoma caeruleum*). Reproductive and agonistic character displacement describes the evolution of mating or fighting traits, respectively, in sympatry between two species in response to selection to avoid maladaptive interspecific interactions. This can result in enhanced behavioral isolation (i.e., preference for conspecific over heterospecific mates) or biases for directing aggressive behaviors toward conspecific over heterospecific rivals in sympatry compared with allopatry (Grether et al. 2009; Pfennig and Pfennig 2012). Although multiple different types of interspecific interactions can generate a pattern of reproductive character displacement (e.g., predation, pollination, parasitoidism, mimicry; reviewed in Hoskin and Higgie 2010) if selection to avoid costly hybridization promotes mating trait divergence in sympatry, reproductive character displacement is equivalent to reinforcement (Pfennig and Pfennig 2012). Under such a scenario, the presence of strong postzygotic barriers causes natural selection to directly favor the evolution of prezygotic barriers, completing the speciation process in sympatry (Coyne and Orr 2004). The relative importance of epistasis, genomic structural variants, and recombination to this mode of speciation remains a major unanswered question.

**Fig. 1.**
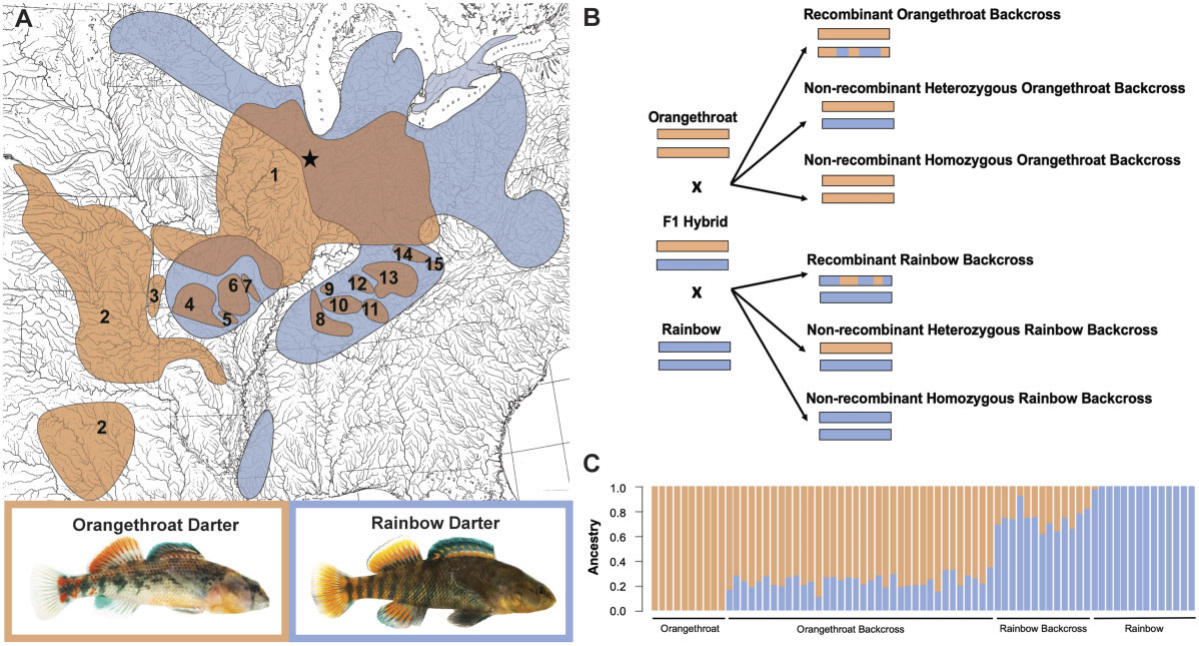
(*A*) Focal species ranges and images. The ranges for each of the 15 species within the orangethroat darter clade are numbered. The approximate collection location for orangethroat darters and rainbow darters used in the present study are marked with a star. (*B*) Schematic depicting crossing design and expected genetic structure for backcrosses to two parental species. Homologous pairs of chromosomes are represented by rectangles. Wild-caught F1 hybrid males were crossed to wild-caught females of both parental species. F1 hybrids are expected to be heterozygous across the genome, with one set of chromosomes from each parental species. In F1s, crossing over during meiosis results in recombinant gametes. In a given chromosomal tetrad, crossing over only occurs in two of the four DNA strands. This predicts that F1 gametes will be 50% recombinant and 50% nonrecombinant (25% orangethroat and 25% rainbow) at each chromosome. Thus, for a given homologous chromosome pair, backcross offspring can have one recombinant and one nonrecombinant chromosome, one chromosome from each parental species (i.e., nonrecombinant heterozygous), or two chromosomes from the same parental species (i.e., nonrecombinant homozygous). (*C*) Individual ancestry proportions for wild-caught orangethroat darters, lab-generated orangethroat backcrosses, lab-generated rainbow backcrosses, and wild-caught rainbow darters. Ancestry proportions were obtained from ADMIXTURE by specifying two ancestral populations.

The orangethroat darter was recently split into a clade of 15 allopatric species (*Ceasia*), 13 of which occur in sympatry with the rainbow darter (fig. 1*A*) (Ceas and Page 1997; Bossu and Near 2009). Although orangethroat and rainbow darters are not sister taxa to one another and are estimated to have diverged 22 Ma (Near et al. 2011), they share similar ecology, mating behavior, and male color patterns (fig. 1*A*) (Page and Burr 2011). F1, F2, and backcross hybrids have been identified in several zones of sympatry between orangethroat and rainbow darters using molecular data (Bossu and Near 2013; Moran et al. 2017, 2018), and Bossu and Near (2013) used microsatellite markers to estimate that 6% of individuals sampled from one sympatric river drainage were admixed. Selection to avoid costly hybridization appears to have promoted increased male mating preferences for conspecific over heterospecific females in sympatry compared with allopatry between these species, consistent with reproductive character displacement (Moran et al. 2017; Moran and Fuller 2018a). Interspecific fighting over access to females has also been documented between male orangethroat and rainbow darters and is likely costly. In turn, selection to avoid interspecific male contests has led to increased male bias for fighting with conspecific over heterospecific males in sympatry compared with allopatry, consistent with agonistic character displacement (Moran et al. 2017; Moran and Fuller 2018a, 2018b).

The genetic basis of postzygotic isolation between orangethroat and rainbow darters remains unknown. As darters provided one of the most compelling examples of how postzygotic isolation promotes reproductive character displacement (which in turn promotes agonistic character displacement) between and within species (Moran et al. 2017, 2018; Moran and Fuller 2018a, 2018b), understanding the mechanism underlying genetic incompatibilities between orangethroat and rainbow darters will inform our understanding of how character displacement evolve. Recent research has shown that lethal genetic incompatibilities are uncovered in the backcross hybrid generation (Moran et al. 2018). These incompatibilities could stem from negative epistatic interactions and/or chromosomal rearrangements. Theory and empirical data suggest that chromosomal inversions may be particularly likely to facilitate the evolution of reproductive character displacement by reducing recombination and preventing the breakup of alleles underlying prezygotic and postzygotic isolation (Noor et al. 2001; Servedio and Noor 2003). There is good reason to suspect that chromosomal rearrangements play a role in postzygotic isolation in darters. Although all species of darters appear to possess 24 pairs of chromosomes, karyotype (e.g., number of metacentric vs. acrocentric chromosomes) can vary within and among species (Moerchen R, unpublished data; Ross 1973; Danzmann 1979). Variation in chromosome morphology is so pervasive in darters that one previous study suggested karyological diversity may reflect species diversity in these fishes (Ross 1973).

Here, we take the first steps toward elucidating the genomic architecture of postzygotic isolation and hybrid incompatibly in darters by evaluating the presence of chromosomal rearrangements and/or negative epistatic interactions between the genomes of orangethroat and rainbow darters. We used both long-read Pacific Biosciences (PacBio) and short-read Illumina sequencing to assemble a genome, transcriptome, and high-density linkage map for the orangethroat darter *Etheostoma* *spectabile*, with the goal of producing a high-quality, annotated genome. To test the hypothesis that chromosomal rearrangements are present between orangethroat and rainbow darters, we also constructed a high-density linkage map for the rainbow darter. This allowed us to compare synteny and homology between rainbow darter and orangethroat darter linkage groups. To further assist in identifying regions of the genome implicated in postzygotic isolation, we conducted fine-scale SNP mapping in wild-caught adults from both parental species and in laboratory-generated backcross individuals (fig. 1*B*). This allowed us to examine genome-wide patterns of selection against recombinant haplotypes, transmission ratio distortion, and linkage disequilibrium in backcross hybrids. This research provides unprecedented insight into the genetic mechanisms promoting the evolution of character displacement. The genomic resources generated by this study will be instrumental for future investigations spanning the fields of speciation, population genomics, conservation, and systematics in darters and other percids.

## Results

### Genome Assembly

We used both Illumina and PacBio sequencing to produce a highly contiguous orangethroat darter genome assembly. We first sequenced two shotgun libraries (450 and 800 bp insert sizes) and three mate-pair libraries (3–5, 5–7, and 8–12 kb insert sizes), which were assembled with Meraculous2 v2.2.2.5 (Chapman JA, Ho IY, Goltsman E, Rokhsar DS, unpublished data). Using an optimal kmer size of 59 (supplementary tables 1 and 2, Supplementary Material online), the Meraculous2 assembly resulted in 4,629 scaffolds >1 kb, a total assembly length of 719.8 Mb with 10.7% gaps, and an N50 of 2.2 Mb. We estimate the total length of the orangethroat darter genome to be ∼1 Gb, based on a *C*-value of 1.06 for another species of darter (the logperch, *Percina caprodes*) (Hardie and Hebert 2004). The Illumina-based Meraculous2 assembly is thus estimated to have a coverage of 145× (supplementary table 3, Supplementary Material online). Additional scaffolding and gap filling of the Illumina assembly was conducted with 30× coverage PacBio reads from a long-insert (>31 kb) library. This provided substantial reduction in gap sizes and increased continuity of the assembly (see table 1 for intermediate assembly statistics), resulting in 3,345 scaffolds >1 kb, a total assembly length of 855.1 Mb with 0.47% gaps, and an N50 of 8.1 Mb. Analysis with BUSCO v3.0.2 (Simao et al. 2015) indicated that 4,314 out of 4,584 total (94.1%) Actinopterygii orthologs were identified as complete in the assembly. Repeat masking with RepeatModeler (v1.0.11; Smit and Hubley 2008) indicated that repetitive elements made up 30.9% (264.2 Mb) of the genome. The GC content of the genome was 40.9%, which is similar to other perciform genomes (e.g., Eurasian perch; Ozerov et al. 2018).

**Table 1.** Summary Statistics for the Orangethroat Darter Genome Assemblies.

Assembly Program	Input Data	No. of Scaffolds	Sequence Total (Mb)	% Gaps	N50 (Mb)	Complete BUSCOs
Meraculous	Illumina mate pair and shotgun reads	4,629	719.8	10.68	2.2	94.5%
PBJelly	Meraculous assembly and PacBio raw reads	3,554	855.2	1.42	3.8	96.3%
wtdgb2	PacBio raw reads	2,593	774.4	0	2.9	95.0%
Canu	PacBio raw reads	6,669	776.2	0	0.4	92.4%
Quickmerge	Canu and wtdbg2 assemblies	4,469	778.2	0	4.5	93.1%
Quickmerge	Canu-wtdbg2 merged assembly and PBJelly assembly	3,345	855.1	0.47	8.1	94.1%
Chromonomer	Canu/wtdbg2/PBJelly merged assembly and linkage map	3,204	855.1 (83% in chromosomes)	0.47	30.5	94.1%

Note.—wtdgb2 discards scaffolds <5 kb in length. The final chromosome-level assembly was produced with Chromonomer.

### Linkage Maps

We constructed linkage maps using RADseq data from a single orangethroat darter family and a single rainbow darter family. RAD sequencing resulted in a mean ± SE depth of coverage per individual of 44 ± 4.33 for the orangethroat linkage map family (2 parents and 145 fry) and 43 ± 1.45 for the rainbow linkage map family (2 parents and 77 fry). Markers clustered into 24 linkage groups in both species. This is in agreement with the number of chromosomes identified in darters previously by karyotyping (Ross 1973; Danzmann 1979). In both orangethroat and rainbow darters, the male parent linkage maps contained fewer loci than the female parent linkage maps. For orangethroat darters, the female linkage map contained 930 markers and was 1,488.89 cM in length and the male linkage map contained 301 markers and was 1,414.41 cM in length. For rainbow darters, the female linkage map contained 991 markers and was 1,804.78 cM in length and the male linkage map contained 744 markers and was 2,137.51 cM in length (table 2). A total of 1,111 markers were incorporated into the final orangethroat darter consensus map (supplementary fig. 2, Supplementary Material online) and 1,616 markers were incorporated into the final rainbow darter consensus map (supplementary fig. 3, Supplementary Material online).

**Table 2.** Summary Statistics for the Sex-Specific and Consensus Linkage Maps for Orangethroat (OT) and Rainbow (RB) Darters.

	OT Female	OT Male	OT Consensus	RB Female	RB Male	RB Consensus
Mean intermaker distance (cM)	1.64	5.11	1.57	1.83	2.97	1.45
Total map length	1,488.89	1,414.41	1,770.27	1,804.78	2,137.51	2,304.04
Mean recombination rate (cM/MB)	2.11	2.00	2.50	2.68	3.15	3.42

### Integrating the Orangethroat Linkage Map and Genome Scaffolds

Out of the 1,111 total markers in orangethroat darter linkage map, 988 had primary alignments to the assembly and were used by Chromonomer v1.08 (http://http://catchenlab.life.illinois.edu/chromonomer/; last accessed November 4, 2019) to join and orient scaffolds into chromosomes. Chromonomer joined and oriented 164 of the 3,345 assembly scaffolds into 24 chromosome-level scaffolds. This resulted in a final assembly with 3,204 scaffolds totaling 855.1 Mb in length, 706.7 Mb (83%) of which were integrated into chromosomes, and an N50 of 30.5 Mb.

### Transcriptome Assembly and Genome Annotation

The orangethroat darter transcriptome was assembled by Trinity v2.5.1 (Grabherr et al. 2011) and contained a total of 366,416 transcripts for 181,974 genes. Analysis with BUSCO indicated that 4,282 out of 4,584 total (93.4%) Actinopterygii orthologs were identified as complete in the transcriptome assembly (supplementary table 4, Supplementary Material online). Maker v2.31.9 (Cantarel et al. 2007) identified a total of 18,867 protein-coding genes, with a mean gene length of 13,747.6 bp. Based on homology with proteins in the UniProt Swiss-Prot database, we were able to assign a putative functional annotation to 18,532 (98.2%) of the orangethroat darter proteins.

### Synteny and Homology Analyses

**Fig. 2.**
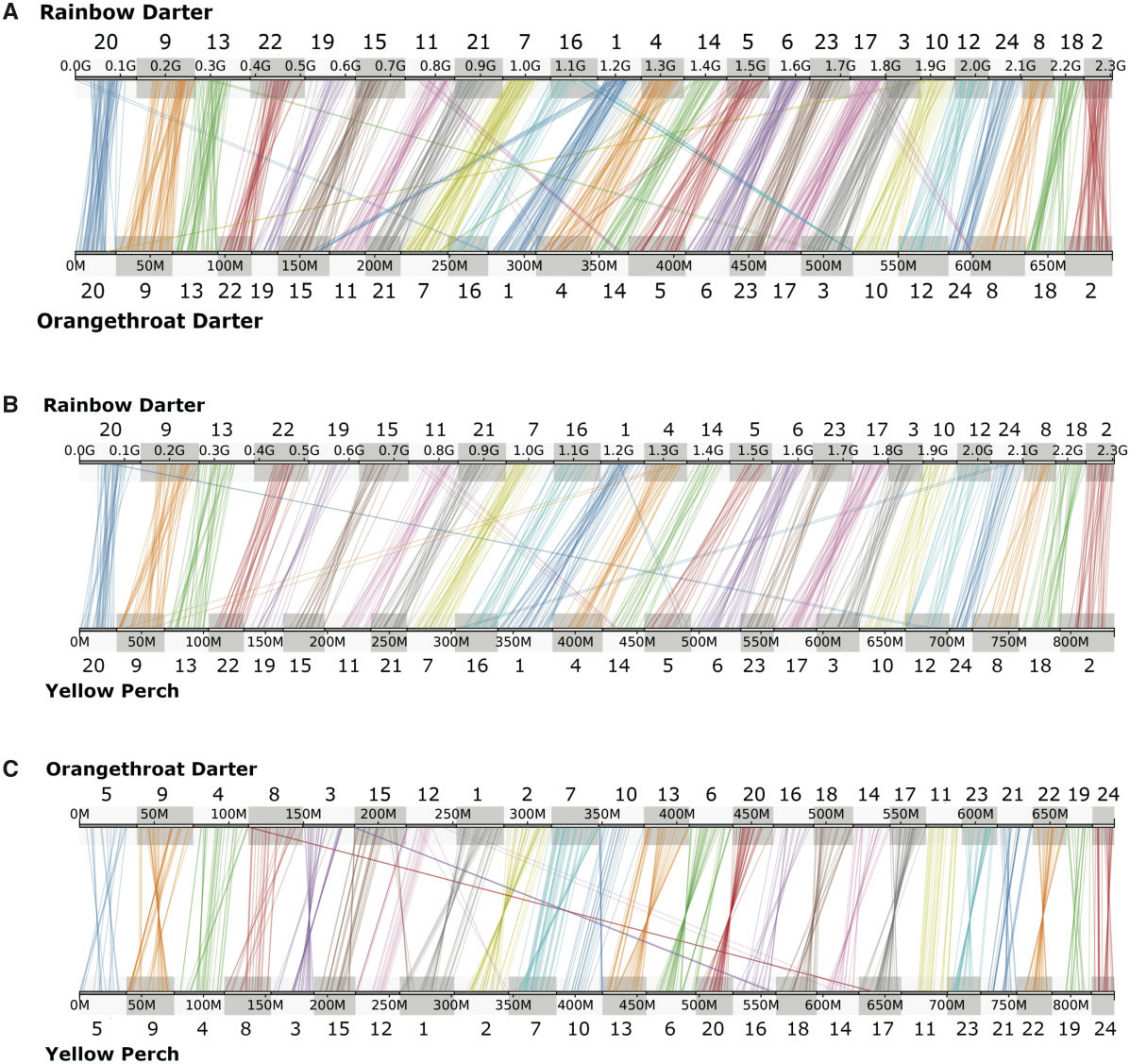
Homology and synteny between linkage groups in the rainbow darter linkage map, orangethroat darter genome assembly, and yellow perch genome assembly. Rainbow darter linkage map markers (i.e., 100 bp RAD tags) were mapped to orangethroat darter (*A*) and yellow perch (*B*) genomes. The locations of these markers were also compared between the orangethroat darter and yellow perch genomes (*C*). Lines represent the alignment position of markers between species pairs, drawn by Synolog using data from reciprocal BLAST searches. Distance along the rainbow darter linkage groups is shown as genetic distance (G) in cM and distance along the orangethroat darter and yellow perch linkage groups is shown in Mb.

To identify any chromosomal rearrangements putatively involved in postzygotic isolation, we compared genomic synteny and homology between the rainbow darter linkage map and the orangethroat darter genome assembly. We also made comparisons between each darter species and a more distant relative within the Percid family, the yellow perch. This outgroup comparison allowed us to infer whether a given rearrangement between the two darter genomes was more likely to have evolved in orangethroat versus rainbow darters. Of the 1,616 total RAD markers included in the final rainbow darter linkage map, Synolog (Small et al. 2016) identified 1,236 aligned to linkage groups in the orangethroat darter genome assembly. A total of 688 of the RAD markers were aligned to both the rainbow darter linkage groups and the yellow perch genome assembly, and 694 of the RAD markers aligned to both the orangethroat darter and yellow perch genome assemblies. We used Synolog to visualize discrepancies in marker grouping and order in each pairwise comparison (fig. 2). We describe the synteny analyses below.

Previous karyotypic analyses have shown that all species in the darter family Percidae have a haploid chromosome number of 24. However, chromosome morphology (e.g., number of metacentric vs. acrocentric chromosomes) typically varies among darter species and even among populations within species, indicating that chromosomal rearrangements may be common in these fishes (Ross 1973; Danzmann 1979). Our results are consistent with these previous karyotype studies. Our synteny analysis revealed a 1:1 homology and conserved sequence order across most of the 24 orangethroat darter and rainbow darter linkage groups (fig. 2*A*). However, we observed differences in the order of RAD tag markers along each homologous pair of orangethroat and rainbow darter linkage groups. This indicates that chromosomal inversions may have occurred multiple times throughout the genome in the time since orangethroat and rainbow darters last shared a common ancestor (fig. 2*A*). Putative translocations were also observed between several linkage group pairs. Well-supported translocations were defined as regions where three or more markers mapped to nonhomologous linkage groups between species. By comparing such translocation events between the two darter species with those observed in comparisons between yellow perch and darters (see below), we were able to infer that translocations between linkage groups 1 and 15, 3 and 16, and 8 and 17 are likely specific to orangethroat darters (i.e., evolved after their split from rainbow darters). Conversely, translocations between linkage groups 4 and 5 and between linkage groups 11 and 14 appear to be specific to rainbow darters.

We observed a 1:1 homologous relationship and conserved sequence order between most rainbow darter and yellow perch linkage groups, although several putative translocations and inversions are evident (fig. 2*B*). Comparing the position of markers in the orangethroat and yellow perch genome assemblies also revealed large stretches of syntenic sequence. Most of the yellow perch linkage groups exhibited 1:1 homology and conserved synteny with the orangethroat darter linkage groups, with the exception of a few apparent translocations and inversions (fig. 2*C*). As the two genomes were assembled independently, the widespread homology we observed with the yellow perch assembly provides a second line of support for the accuracy of the orangethroat darter assembly.

### Identifying Putative Genetic Incompatibilities Using Backcross Genomes

To further assist in identifying regions involved in postzygotic isolation between orangethroat and rainbow darters, we examined genome-wide patterns of local ancestry in lab-generated backcross hybrids with RADseq. We first used ADMIXTURE (v1.3.0) (Alexander et al. 2009) to verify that ancestry proportions were in accordance with expectations for backcross individuals. As expected, the mean ± SE proportion of orangethroat darter ancestry in backcrosses to orangethroat darters was 0.75 ± 0.01 (*n* = 36), and the mean ± SE proportion of rainbow darter ancestry in backcrosses to rainbow darters was 0.73 ± 0.02 (*n* = 13) (fig. 1*C*). We then used both a haplotype-based and a SNP-based approach to ask whether certain regions of the genome showed significant deviations from expected ancestry proportions consistent with selection against hybrid incompatibilities. The results of these analyses are detailed below.

#### Mapping Local Ancestry across Backcross Hybrid Genomes

**Fig. 3.**
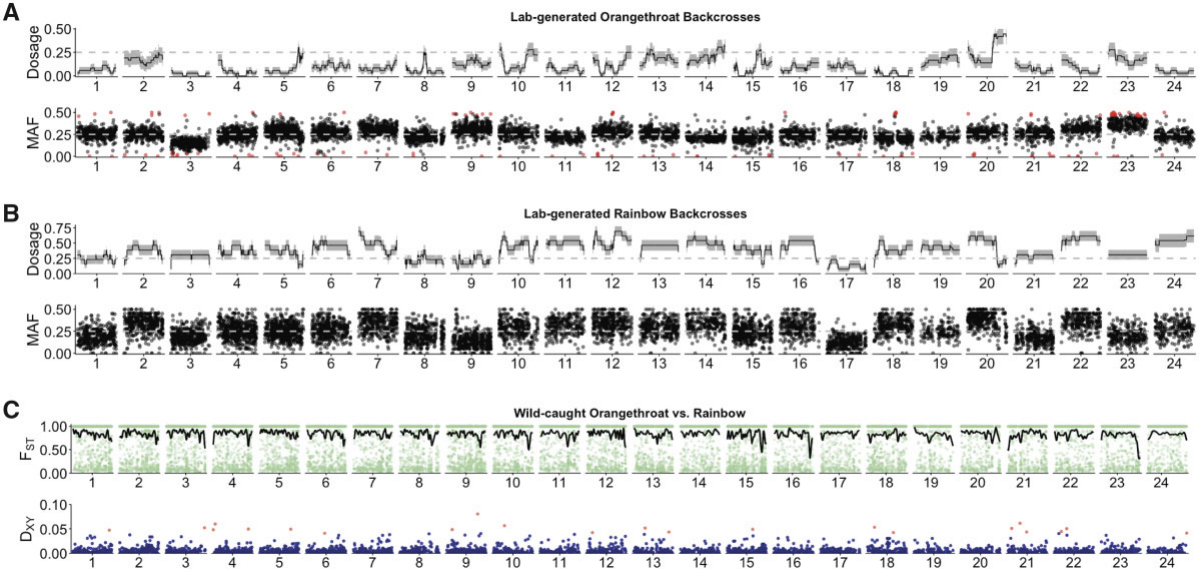
Mean minor parent allele dosage (“Dosage”) and minor allele frequency (“MAF”) along each linkage group for (*A*) 36 orangethroat backcross fry and (*B*) 13 rainbow backcross fry. The minor parent allele dosage was calculated using a Hidden Markov Model implemented in ELAI. ELAI inferred ancestry along each linkage group for each backcross fry using a set of 29,064 SNPs. In backcrosses, the minor parent allele dosage can range from 0.0 to 1.0 for a given linkage group (see fig. 1*B*), and the mean minor parent allele dosage is expected to be 0.25. An allele dosage of 1.0 indicates perfect heterozygosity, with one allele originating from the minor parent and the other from the major parent. An allele dosage of 0.0 represents nonadmixed regions of the genome with zero minor parent alleles (and two major parent alleles). SE around the mean allele dosage is shown in gray. MAF is shown for a set of 8,177 SNPs. SNPs that deviate significantly from the expected MAF of 0.25 are shown in red (5% FDR). (*C*) *F*_ST_ and *D*_XY_ were calculated between 10 wild-caught orangethroat darters and 14 wild-caught rainbow darters at 43,502 SNPs across the genome. *F*_ST_ for the 39,518 SNPs that mapped to 1 of the 24 linkage groups is shown in green. Smoothed *F*_ST_ (black line) was calculated in sliding 500 kb windows. *D*_XY_ was calculated in nonoverlapping 50 kb windows, resulting in a total of 3,443 windows covering 0.81 million sites across the genome. *D*_XY_ windows mapping to 1 of the 24 linkage groups (3,062 total) are shown, with outlier windows above the 99th percentile (*D*_XY_>0.0407) in red.

Here, we used a haplotype-based approach to investigate patterns of local ancestry in backcross offspring. Specifically, we used RADseq data from nonintrogressed, wild-caught orangethroat and rainbow darters to train a two-level Hidden Markov Model in ELAI v1.00 (Guan 2014). We then used the model to infer switches in local ancestry (i.e., recombination breakpoints) from homozygous (nonintrogressed, nonrecombinant) to heterozygous (introgressed, recombinant) regions across backcross hybrid genomes (see supplementary fig. 4, Supplementary Material online, for representative examples of recombinant and nonrecombinant linkage groups). The mean ± SE minor parent allele dosage along each linkage group for both sets of backcross fry is shown in figure 3*A* and *B*.

**Fig. 4.**
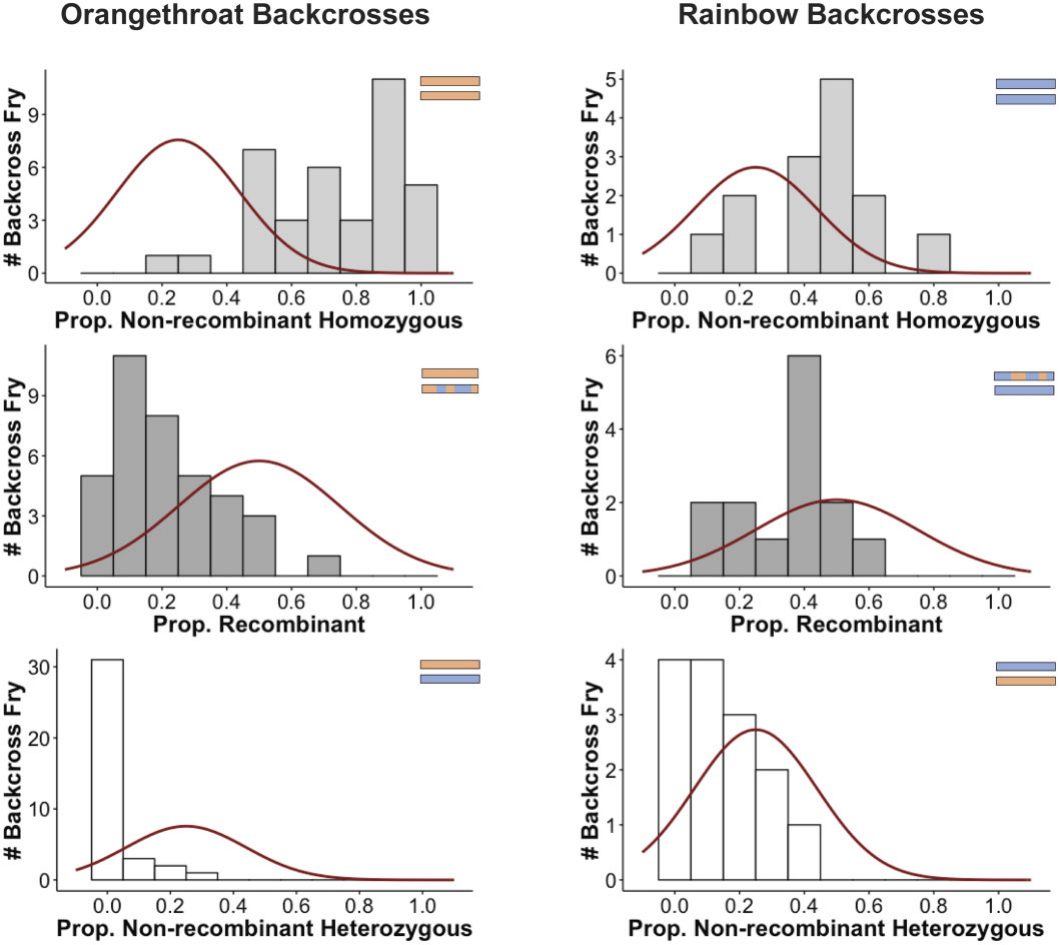
Distributions of the proportion of nonrecombinant homozygous, recombinant, and nonrecombinant heterozygous linkage groups (see fig. 1*B*) in backcrosses to orangethroat darters (left column; *n* = 36 fry) and in backcrosses to rainbow darters (right column; *n* = 13 fry). The expected distribution is overlaid in red.

In hybrid genomes, selection and recombination are expected to generate a pattern of enrichment for major parent ancestry (i.e., decreased heterozygosity) at regions associated with genetic incompatibilities between parental species (Barton and Hewitt 1985; Rieseberg et al. 1999; Turelli et al. 2001; Nachman and Payseur 2012; Schumer et al. 2018). We asked whether backcross offspring deviated from the expected 50% recombinant, 25% homozygous (both chromosomes inherited from the same parental species), and 25% heterozygous (one chromosome inherited from each parental species) across all 24 linkage groups (fig. 1*B*). For each linkage group in both backcross directions, we counted how many offspring showed each of the three possible types of chromosome pairs. We observed a lower than expected number of recombinant haplotypes in backcrosses to orangethroat darters (χ^2^ = 157.22, d.f. = 23, *n* = 36, *P* < 0.0001) but not in backcrosses to rainbow darters (χ^2^ = 31.39, d.f. = 23, *n* = 13; *P* = 0.11). Similarly, heterozygous haplotypes were lower than expected in backcrosses to orangethroat darters (χ^2^ = 914.67, d.f. = 23, *n* = 36, *P* < 0.0001) but not in backcrosses to rainbow darters (χ^2^ = 30.31, d.f. = 23, *n* = 13, *P* = 0.19). There was an enrichment of homozygous haplotypes in both backcrosses to orangethroat darters (χ^2^ = 174.22, d.f. = 23, *n* = 36, *P* < 0.0001) and in backcrosses to rainbow darters (χ^2^ = 93.69, d.f. = 23, *n* = 13, *P* < 0.0001). Similar observations were found when examining individual linkage groups (fig. 4 and supplementary tables 5 and 6, Supplementary Material online).

#### Deviations from Mendelian Segregation in Backcrosses

Regions in hybrid genomes that deviate from the expected Mendelian segregation ratios (also called transmission ratio distortion; Hall and Willis 2005) have been linked to genetic incompatibilities stemming from chromosomal rearrangements and negative epistatic interactions (Rieseberg et al. 1995; De Villena and Sapienza 2001; Leppälä et al. 2013). Here, we identified a set of 8,177 ancestry-informative SNPs that were differentially fixed in wild-caught orangethroat and rainbow darters. We calculated allele frequencies at these sites in both sets of backcross offspring to quantify patterns of transmission ratio distortion (i.e., deviation from the expected MAF of 0.25) across the genome. The mean ± SE MAF in backcrosses to orangethroat darters was 0.254 ± 0.001. The mean ± SE MAF in backcrosses to rainbow darters was 0.258 ± 0.001. We expect reduced heterozygosity in regions associated with genetic incompatibilities, and both positive and negative distortions can achieve this effect. The magnitude of transmission ratio distortion varied between the two backcross directions. After applying a 5% FDR correction to account for multiple tests, we observed that 115 (1.4%) out of 8,177 total SNPs deviated significantly from expected frequencies in the fry resulting from backcrosses between F1 hybrid males and female orangethroat darters (fig. 3*A*). The mean ± SE number of SNPs significant deviating from the expected MAF was 4.80 ± 0.85 across all 24 linkage groups and ranged from 20 deviating SNPs on linkage group 23 to 0 deviating SNPs on linkage group 19.

After applying a 5% FDR correction, none of 8,177 total SNPs deviated significantly from expected frequencies in the fry resulting from backcrosses between F1 hybrid males and female rainbow darters (fig. 3*B*). This may be attributable to the fact that we had lower power to detect transmission ratio distortion in the backcrosses to rainbow darters (*n* = 13 fry) compared with the backcrosses to orangethroat darters (*n* = 36 fry).

### Patterns of Recombination Rate Variation across Parental Genomes

To estimate local recombination rate across the genome separately in orangethroat and rainbow darters, we supplied a set of 29,064 SNPs present in both species (see Materials and Methods) to the Interval program in LDhat v2.2 (McVean and Auton 2007). The population-level recombination rate, rho (*ρ = * 4 *N*_e_*r*), was similar for both orangethroat and rainbow darters (orangethroat darters: mean ± SE *ρ  = * 0.93 ± 0.01; rainbow darters: mean ± SE *ρ  = * 0.88 ± 0.03). In both species, most linkage groups showed at least one region of suppressed recombination (i.e., a plateau at *ρ  = * 0), potentially corresponding to centromere location (supplementary figs. 5 and 6, Supplementary Material online). Recombination rate varied between *ρ = * 0–8 orangethroat darters and *ρ = * 0–25 in rainbow darters. In orangethroat darters, extreme recombination rate peaks were observed on linkage groups 1 and 21 (supplementary fig. 5, Supplementary Material online). In rainbow darters, extreme recombination rate peaks were observed on linkage groups 1, 2, 4, 9, 13, 14, 18, and 21 (supplementary fig. 6, Supplementary Material online). These peaks may indicate regions harboring chromosomal rearrangements relative to the reference genome (which may explain the higher incidence of hotspots in rainbow darters relative to orangethroat darters) or mis-assemblies in the reference genome. We excluded extreme values of *ρ* >2, which are not likely biologically relevant, for subsequent correlation analyses with *D*_XY_ (see below).

### Genetic Differentiation between Species

We assessed genome-wide levels of divergence between orangethroat and rainbow darters to identify any outlier regions potentially associated with reproductive isolation. We first calculated *F*_ST_ between 10 wild-caught orangethroat darter and 14 wild-caught rainbow darters at 43,502 SNPs across 16,950 RAD loci (∼1.6 million sites) and calculated smoothed *F*_ST_ in 500 kb sliding windows. In total, 39,518 SNPs mapped to 1 of the 24 linkage groups. Despite ongoing hybridization, *F*_ST_ between orangethroat and rainbow darters was generally high across the genome (fig. 3*C*), with 31,940 out of 43,502 SNPs (73%) fixed between species (genome-wide mean *F*_ST_ = 0.83). Because relative measures of genetic divergence such as *F*_ST_ are sensitive to levels of genetic diversity within species, we also calculated an absolute metric of divergence, *D*_XY,_ in 50 kb nonoverlapping windows across the genome. This resulted in 3,443 windows and included 0.81 million sites. The mean genome-wide *D*_XY_ between orangethroat and rainbow darters was 0.0068. Of the 3,062 *D*_XY_ windows mapped to a linkage group, we identified 22 outlier windows (i.e., regions above the 99th percentile, 0.0407) spread across 14 linkage groups (fig. 3*C*). Thus, outlier regions with high absolute genetic divergence do not appear to be localized to just one or a few genomic regions.

Genomic regions with reduced recombination rate are predicted to have increased genetic divergence between species and may harbor alleles important to reproductive isolation (Albert and Schluter 2004; Nachman and Payseur 2012; Ortiz-Barrientos et al. 2016; Meier et al. 2018). In orangethroat darters, we observed a negative correlation between *D*_XY_ and the population-level recombination rate, *ρ* (*n* = 3,037, *r* = −0.14, *P* < 0.00001). Conversely, we did not observe any correlation between *D*_XY_ and *ρ* in rainbow darters (*n* = 3,037, *r* = 0.01, *P* = 0.47). The fact that *ρ* is affected by demographic processes may account for the difference in the relationship between *D*_XY_ and *ρ* observed between the two species, as rainbow darters typically have larger effective population sizes relative to orangethroat darters (see below).

Regions of the genome important to reproductive isolation are expected to exhibit high levels of divergence between species and transmission ratio distortion in hybrid genomes. Consistent with this prediction, Pearson correlation indicated a significant positive relationship between *D*_XY_ between orangethroat and rainbow darters and deviation from the expected MAF in backcrosses to orangethroat darters (*n* = 3,037, *r* = 0.09, *P* < 0.00001). We did not observe any correlation between *D*_XY_ and MAF in backcrosses to rainbow darters (*n* = 3,037, *r* = −0.01, *P* = 0.67).

We observed that nucleotide diversity calculated with variant and invariant sites is higher in rainbow darters (π = 0.0017) compared with orangethroat darters (π = 0.0011) (supplementary fig. 7, Supplementary Material online). This finding is in agreement with previous studies in this system (Moran et al. 2017, 2018) and likely reflects ecological differences between these species. Larger order streams and rivers typically act as an impediment to gene flow among orangethroat darter populations, but not among rainbow darter populations (Page 1983), which likely contributes to higher nucleotide diversity in rainbow darter populations relative to orangethroat darter populations.

### Gene Ontology Enrichment Analysis

We used the orangethroat darter genome annotation to identify a total of 44 genes associated with *D*_XY_ outlier regions (supplementary table 7, Supplementary Material online). PANTHER (v14.1) (Huaiyu et al. 2019) identified 149 significantly enriched GO terms associated with these genes (supplementary table 8, Supplementary Material online), which were condensed down to 39 nonredundant GO terms by REVIGO (Supek et al. 2011). Notably, some of the most overrepresented ontologies were related to developmental processes (e.g., development of the nervous system, blood vessels, skeletal muscle, and bones), chromosome organization, and regulation of meiotic processes related to sister chromatid segregation (supplementary table 9, Supplementary Material online). Other overrepresented ontologies included methylation and epigenetic gene expression, neuron differentiation, detection of stimulus involved in sensory perception, and regulation of hormone receptor signaling pathways (supplementary table 9, Supplementary Material online).

## Discussion

Here, we presented the first reference genome, transcriptome, and linkage maps for darters, the most diverse group of vertebrates in North America. We produced a highly contiguous, chromosome-level annotated assembly of the orangethroat darter genome by combining Illumina and PacBio whole-genome sequencing with a high-density linkage map (table 1). By generating a linkage map for the rainbow darter, we were also able to compare genomic synteny and homology between orangethroat and rainbow darters (estimated divergence 22 Ma), and between both darters and an outgroup, the yellow perch (estimated divergence 58–60 Ma). Additionally, we used RADseq to genotype 1) nonadmixed individuals from natural populations of orangethroat and rainbow darters and 2) individuals produced from laboratory-generated backcrosses between wild-caught F1 hybrid males and females of both parental species. We conducted fine-scale ancestry mapping in backcross hybrid genomes and identified patterns consistent with wide-spread genetic incompatibilities and strong selection against recombinant haplotypes. We also observed that outlier regions with high levels of genetic divergence between parental species were enriched for genes associated with developmental and meiotic processes, providing candidates for postzygotic isolating barriers. Together, our analyses of the annotated darter genome assembly, linkage maps, and RADseq data provide new insights into the genomic architecture of postzygotic isolation in naturally hybridizing species undergoing reproductive and agonistic character displacement.

### Genomic Patterns of Divergence between Orangethroat and Rainbow Darters

We generally observed 1:1 homology and conserved synteny between the rainbow darter linkage map, orangethroat darter genome, and yellow perch genome. However, several putative chromosomal rearrangements were observed in all comparisons (fig. 2), which may play a role in conferring hybrid incompatibility (see below). We detected widespread genomic divergence between orangethroat and rainbow darters, implying that numerous regions across the genome act as barriers to gene flow. *D*_XY_ outlier regions were observed across 14 linkage groups and *F*_ST_ was surprisingly high across the entire genome given the presence of ongoing hybridization. These results suggest that “islands” of genomic divergence are not localized at a few discrete genomic regions (Turner et al. 2005; Harr 2006; Nosil et al. 2009). Similar patterns of genome-wide divergence despite ongoing gene flow have also been observed in *Anopheles* mosquitoes (Lawniczak et al. 2010), threespine stickleback (Hohenlohe et al. 2010; Roesti et al. 2012), cichlids (Meier et al. 2018), and *Drosophila* (McGaugh and Noor 2012). Theory suggests that widespread divergence can evolve rapidly even in the face of gene flow when selection is acting on multiple loci throughout the genome, sensu “multifarious selection,” in conjunction with genomic hitchhiking (Rice and Hostert 1993; Feder and Nosil 2010). Such a scenario may be most likely to occur with secondary contact (Barton and Hewitt 1985; Barton and Bengtsson 1986). There is good reason to suspect that orangethroat and rainbow darters initially diverged in allopatry followed by a secondary contact event, as orangethroat and rainbow darters are not sister taxa (Near et al. 2011) and speciation appears to be initiated in allopatry in darters (Near and Benard 2004; Hollingsworth and Near 2009). In addition to selection against genetic incompatibilities, strong selection for enhanced prezygotic isolation in sympatry via reproductive and agonistic character displacement has likely also played a large role in promoting genomic divergence between orangethroat and rainbow darters (Moran and Fuller 2018a, 2018b). Examining genomic divergence between multiple sympatric and allopatric population pairs of orangethroat and rainbow darters may help to distinguish regions of the genome under selection due to reproductive and agonistic character displacement versus neutral regions that have accumulated divergence in allopatry.

### Synthesizing Patterns Associated with Genetic Incompatibilities

Multiple linkage groups appear to harbor genetic incompatibilities implicated in postzygotic isolation between orangethroat and rainbow darters. The genomes of viable backcross fry showed a striking bias for homozygous ancestry from a single parental species (fig. 4). The lack of recombinant haplotypes and presence of alleles under transmission ratio distortion was enhanced in backcrosses to orangethroat darters relative to backcrosses to rainbow darters (figs. 3*A* and *B* and 4). Putative translocations supported by three or more markers were observed across ten linkage groups (fig. 2). Notably, linkage groups 1, 4, and 14 show evidence of a translocation between orangethroat and rainbow darters and also exhibit regions of increased recombination rate relative to the background level in rainbow darters, which further supports that these chromosomes contain rearrangements (supplementary fig. 6, Supplementary Material online). This observation is in agreement with a previous study of chromosome morphology that identified karyotypic differences between these species (Moerchen R, unpublished data).

The hypothesis that structural differences underlie postzygotic isolation predicts transmission ratio distortion and a reduction in recombinant haplotypes at linkage groups exhibiting rearrangements between orangethroat and rainbow darters. We observed that structural rearrangements between and within linkage groups appear to be pervasive. Deviations from conserved synteny along homologous linkage groups indicative of putative chromosomal inversions were observed within each of the 24 linkage groups. Inversions are commonly implicated in suppressed recombination between and within species, and may play a particularly important role in speciation with gene flow (Rieseberg et al. 1999; Noor et al. 2001; Rieseberg 2001; McGaugh and Noor 2012). Although there was no obvious association between the location of SNPs exhibiting statistically significant levels of transmission ratio distortion in backcrosses and putative rearrangements, regions with higher genetic divergence between the parental species contained SNPs with higher levels of transmission ratio distortion and had a reduced local recombination rate (see below). Considered together with previous reports of differences in chromosome morphology between orangethroat and rainbow darters, our results provide strong evidence that chromosomal rearrangements are common and contribute to postzygotic isolation between these species, which in turn promotes the evolution of prezygotic isolation via reproductive and agonistic character displacement (Moran et al. 2017; Moran and Fuller 2018a). Studies of the genomic architecture of character displacement have been limited, but previous theoretical and empirical work suggests that inversions could facilitate the evolution of reproductive character displacement between hybridizing species by maintaining linkage between loci conferring prezygotic and postzygotic isolation (Noor et al. 2001; Servedio and Noor 2003). Here, we demonstrate that chromosomal inversions themselves likely constitute postzygotic barriers in a system where speciation has proceeded via reproductive and agonistic character displacement.

Genomic regions exhibiting high levels of genetic divergence between hybridizing species are predicted to harbor genes important to reproductive isolation (Feder et al. 2012). The accumulation and maintenance of genetic divergence in the face of gene flow may be facilitated by local reductions in recombination rate near genes that are important to reproductive isolation. Genomic regions with reduced recombination compared with the background level are more likely to be resistant to gene flow between hybridizing species, allowing processes such as drift and selection to drive genetic divergence (Bürger and Akerman 2011; Ortiz-Barrientos et al. 2016). We observed a significant positive correlation between regions of heightened genetic divergence (*D*_XY_) between orangethroat and rainbow darters and regions exhibiting high levels of transmission ratio distortion in orangethroat backcross offspring. We also observed a significant negative correlation between genetic divergence between orangethroat and rainbow darters and the population-level recombination rate in orangethroat darters. Notably, our GO analysis revealed that *D*_XY_ outlier regions exhibiting extreme levels of genetic differentiation between orangethroat and rainbow darters were enriched for genes related to developmental processes, gene expression, and chromosomal segregation and sister chromatid pairing during meiosis (supplementary tables 7–9, Supplementary Material online). Given the high levels of backcross embryo mortality, apparent strong selection against recombinant backcross haplotypes, and evidence of chromosomal rearrangements that we presented here, these genes are likely candidates for postzygotic isolating barriers between orangethroat and rainbow darters. Genes related to transcriptional regulation and chromosome segregation have been shown to underly postzygotic isolation in several other taxa (reviewed in Mack and Nachman 2017), including *Drosophila* (Barbash et al. 2003; Thomae et al. 2013) and mice (Mihola et al. 2009). *D*_XY_ outlier windows were also enriched for genes involved in neuron differentiation, detection of stimulus involved in sensory perception, and response to steroid hormones (supplementary tables 7–9, Supplementary Material online). These genes will serve as candidates for future studies investigating the genomic basis of signals and preferences associated with behavioral isolation and character displacement in this system.

We note that a limitation of our study was that the majority of backcross offspring in both cross-directions were sired by one F1 hybrid male (35 out of 36 offspring in backcrosses to orangethroat darters; 10 out of 13 offspring in backcrosses to rainbow darters). Thus, the reduction in recombinant haplotypes that we observed in backcross offspring may be due to some intrinsic attribute specific to this F1 male that suppressed crossing over during meiosis. However, the overall qualitative patterns appear to hold in the few offspring sired by two other F1 hybrid males. Our low sample size for backcrosses to rainbow darters (13 offspring total) may have also affected our ability to detect negative genetic interactions. These low sample sizes were unavoidable as the overall survival rate was low in back crosses (7%) compared with parental clutches (65%) (Moran et al. 2018).

### Conclusions

The annotated orangethroat darter genome, linkage maps for orangethroat and rainbow darters, and fine-scale genomic data for backcross hybrids presented here have provided an unprecedented insight into the genomic architecture and distribution of postzygotic barriers in darters. Notably, this study represents one of the few investigations to date to characterize genome-wide patterns of hybrid incompatibilities in a long-lived, nonmodel species and in a system where character displacement drives speciation. The presence of numerous chromosomal rearrangements and an enrichment of parental genotypes and transmission ratio distortion across hybrid genomes indicates that genetic incompatibilities are widespread rather than localized to a few regions. Our results suggest that chromosomal rearrangements may contribute to hybrid inviability and thus maintain strong postzygotic isolation between orangethroat and rainbow darters, despite the occurrence of viable, fertile F1 hybrids in natural populations (Moran et al. 2018). The low abundance of recombinant haplotypes across hybrid genomes suggests that genetic incompatibilities are pervasive and fuel selection against hybridization, which may in turn favor the evolution of strong prezygotic barriers in sympatry between orangethroat and rainbow darters via reproductive and agonistic character displacement (Moran et al. 2017; Moran and Fuller 2018a, 2018b). Our findings contrast those of several previous studies in this system that concluded postzygotic isolation is likely an insignificant barrier to gene flow between congeneric darter species (Hubbs and Strawn 1957; Hubbs 1959). The genomic tools generated here will undoubtedly facilitate future studies aimed at examining the genomics of speciation, sexual selection, and ecological adaptation in this highly diverse group of fishes and will provide the opportunity to further develop darters into a model system for studying the genomics of speciation via character displacement (Moran et al. 2017, 2018; Moran and Fuller 2018a, 2018b). Lastly, we anticipate that the darter genome will constitute a valuable resource for conservation efforts. Darters are highly sensitive to anthropogenic disturbances (Albritton 1994; Juracek et al. 2017), and nearly half of all species within the Percid family are considered imperiled (Helfman et al. 2009). Having a high-quality genome available will open the door to future studies aimed at quantifying and preserving genomic variation in populations of conservation concern (Fitzpatrick et al. 2014; Juracek et al. 2017).

## Materials and Methods

### Linkage Map Sequencing

#### Orangethroat Darter

We used a single male and female pair of orangethroat darters to create an F1 mapping cross. Both orangethroat darter parents were collected via kick seine from the Salt Fork of the Vermillion River Drainage (Champaign County, Illinois) in May 2016 (fig. 1*A*). The male parent used in this cross was the same individual whose DNA was used to sequence all genomic libraries described below (i.e., Illumina shotgun, Illumina mate-pair, and PacBio). The pair were housed in the laboratory in a 37 L aquarium with gravel substrate and allowed to spawn. After 24 h, the gravel was siphoned and a total of 176 eggs were collected. Eggs were maintained as described in Moran et al. (2018) until 2-month posthatching. A total of 145 fry survived to this age. At this time, the 2 parents and 145 offspring were euthanized with an overdose of buffered MS-222, placed in 95% ethanol, and stored at −30 °C.

We isolated DNA from the white muscle tissue of both parents and the entire body of each fry. DNA was extracted using a modified Puregene (Qiagen; www.qiagen.com) protocol (Berdan et al. 2014) and treated with RNase A. Samples were checked for purity on a Nanodrop 1000 machine and quantified on a Qubit fluorometer. Restriction site associated DNA sequencing (RADseq) libraries were constructed at Floragenex (Portland, OR) following Baird et al. (2008). The restriction enzyme *SbfI* was used to digest 750 ng of DNA from each of the progeny. To ensure that diploid genotypes were accurately called at each RAD locus for the two parents, 1.5 μg of DNA was included for both parents in the RADseq libraries. Libraries were sequenced as 1×100 bp reads on two lanes on an Illumina HiSeq4000 machine at the University of Oregon (Eugene, OR).

#### Rainbow Darter

To allow for an investigation of genomic synteny between orangethroat and rainbow darters, we also created an F1 mapping cross with a single rainbow darter pair. Both parents were collected via kick seine from the Salt Fork of the Vermillion River Drainage (Champaign County, Illinois) in April 2018. The pair were allowed to spawn in the laboratory and eggs were collected as described earlier. Out of 106 total eggs collected, 85 survived to 2-month posthatching. At this time, the parents and fry were euthanized and DNA was extracted as described earlier. A RAD library was constructed using 1,000 ng of DNA from each of the parents and offspring. We used the restriction enzyme *SbfI* for RAD library construction at the University of Illinois at Urbana-Champaign (UIUC), following the methodology of Baird et al. (2008). We again ensured a higher depth of coverage for the parents by including 2× the amount of DNA for both parents compared with the fry in the RADseq library. This library was sequenced as 1×100 bp reads on two rapid-run lanes on an Illumina HiSeq2500 machine at the Biotechnology Center at UIUC.

### Linkage Map Construction

We used the Stacks (v1.48; Catchen et al. 2011, 2013) program *process_radtags* to demultiplex the raw sequences resulting from the linkage map RADseq libraries (235,042,086 orangethroat darter raw reads; 185,333,523 rainbow darter raw reads) and to remove barcodes and low-quality reads. Eight rainbow darter fry were removed from all further analyses due to a low number of reads, which left 77 rainbow darter fry total. The reads that were retained after the initial quality filtering step (232,255,220 orangethroat darter retained reads; 171,768,983 rainbow darters retained reads) were supplied to the Stacks *denovo_map* pipeline for RAD loci assembly and genotyping for each of the two linkage map families. A minimum of three identical reads (-m 3) were required to form a “stack” (i.e., a putative allele) in each individual. We allowed for a maximum of five differences between stacks to form a locus (-M 5) and a maximum of three differences when merging loci from different individuals to form a catalog (-*n* 3) (Small et al. 2016).

Constructing a linkage map from a single F1 cross requires an identification of polymorphisms that are present in the parents. SNP markers that are informative for mapping in this context include those that are heterozygous in the male parent and homozygous in the female parent (*lm*×*ll*, segregating 1:1), heterozygous in the female parent and homozygous in the male parent (*nn*×*np*, segregating 1:1), heterozygous in both parents with two shared alleles (*hk*×*hk*, segregating 1:2:1), or heterozygous in both parents with one shared allele and one allele specific to each parent (*ef*×*eg*, segregating 1:1:1:1) (Amores et al. 2011). Stacks was used to genotype our linkage map parents and their progeny at each RAD locus. This resulted in 3,478 genotyped loci in the orangethroat darter family and 4,665 genotyped loci in the rainbow darter family that were informative for mapping. For both the orangethroat and the rainbow families, we used the *genotypes* program in Stacks to filter loci for quality and export the resulting data in Cross Pollinator format. Due to higher coverage in the rainbow library compared with the orangethroat library (see Results), we specified that a locus had to be present in at least 30 out of the 145 total orangethroat darter offspring and in at least 45 out of the 77 total rainbow darter offspring to be included in the exported files. This resulted in 2,247 orangethroat darter loci and 3,230 rainbow darter loci that were then imported into JoinMap v5 (Van Ooijen 2006). For each species, linkage maps were constructed separately for both parents. Linkage groups with more than two loci shared between the male and female parent were inferred to be homologous and were combined to form a consensus map. Loci with segregation frequencies that differed significantly from Hardy–Weinberg equilibrium (*P* < 0.001) were excluded. Markers were assigned to linkage groups using an LOD of 4.0 for the orangethroat family and 5.0 for the rainbow family; again, these differences between the species were due to higher coverage in the rainbow darter library. Ungrouped loci were iteratively added to linkage groups by using the Strongest Crosslinked Loci (SCL) option in JoinMap with an LOD cutoff of 4.0. Marker order for each linkage group was calculated using the Kosambi mapping function in JoinMap, which converts recombination frequencies between pairs of markers into genetic distance in cM.

### Genome Sequencing

#### Illumina Paired-End and Mate-Pair Short-Read Libraries

Using standard ethanol precipitation methods, we isolated 19 μg of high-molecular weight DNA from a single wild-caught male orangethroat darter (location details described earlier). This same individual was used as the male parent in the orangethroat darter linkage map cross. Two genomic shotgun libraries with insert sizes of 450 and 800 bp, and three mate-pair libraries (3–5, 5–7, and 8–12 kb) were prepared and sequenced at the Biotechnology Center at UIUC. Shotgun libraries were prepared with the Hyper Library construction kit from Kapa Biosystems. Mate-pair libraries were constructed with the Nextera Mate Pair library Sample Prep kit (Illumina, CA), followed by the TruSeq DNA Sample Prep kit. Libraries were quantitated with qPCR prior to sequencing.

We sequenced the 450-bp shotgun library together with the RNAseq library (see below for details) on two lanes on a HiSeq2500 machine in a proportion of 3:1 (favoring coverage for the shotgun library) to produce paired-end 250 bp reads. Fragment sizes ranged from 200 to 530 bp with an average of 450 bp. We sequenced the 800-bp library and the mate-pair libraries together on one lane for 161 cycles from each end of the fragments on a HiSeq2500 machine, resulting in 150 bp paired-end reads. Fragment sizes ranged from 600 to 900 bp with an average of 800 bp.

Sequencing resulted in a total of 391,068,018 overlapping raw reads from the 450 bp insert library, 63,746,270 raw reads from the 800 bp insert library, 97,973,478 raw reads from the 3–5 kb mate-pair library, 91,585,478 raw reads from the 5–7 kb mate-pair library, and 90,833,126 raw reads from the 8–12 kb mate-pair library. In total, this represents 149× predicted coverage of the 1 Gb genome (Hardie and Hebert 2004). For each library, fastq files were generated and demultiplexed with the bcl2fastq v2.17.1.14 Conversion Software (Illumina).

#### PacBio Long-Read Library

We isolated a total of 40 μg of high-molecular weight DNA from the same male orangethroat darter used in the Illumina shotgun, mate-pair, and RAD libraries. A PacBio long-insert (>31 kb) library was constructed following standard protocol. Sequencing was conducted on four SMRT cells on a PacBio Sequel machine. Library construction and sequencing were both carried out at the University of Minnesota Genomics Center (St. Paul, MN). A total of 30 Gb of raw sequence data (30× genome coverage) was produced, with a mean ± SE longest subread length of 8.4 ± 0.3 kb and a mean ± SE longest subread N50 of 14.6 ± 0.4 kb.

### Genome Assembly

We used the program *process_shortreads* in Stacks to remove adaptors and poor-quality reads from the Illumina shotgun libraries. NxTrim was used to remove biotin adaptors and poor-quality reads from the three Illumina mate-pair libraries. After this quality filtering, we retained a total of 388,991,066 overlapping paired-end reads from the 450 bp insert library, 63,200,545 nonoverlapping paired-end reads from the 800 bp insert library, 79,139,172 3–5 kb mate-pairs, 76,273,856 5–7 kb mate-pairs, and 78,571,066 8–12 kb mate-pairs. This represents 145× coverage of the ∼1 Gb orangethroat darter genome. We used Meraculous2 v2.2.2.5 (Chapman JA, Ho IY, Goltsman E, Rokhsar DS. unpublished data.) to carry out four de novo genome assemblies with kmer length of 49, 59, 69, and 79. We obtained assembly statistics from Meraculous2 and QUAST v4.4 (Gurevich et al. 2013) (supplementary table 1, Supplementary Material online) and examined the number of Actinopterygii-specific Benchmarking Universal Single-Copy Orthologs (BUSCOs) identified in each assembly with BUSCO v3.0.2 (Simao et al. 2015) (supplementary table 2, Supplementary Material online). A kmer size of 59 yielded the best assembly based on quality and completeness (supplementary tables 1 and 2, Supplementary Material online).

We took two different approaches to improve the Illumina-based assembly obtained from Meraculous2 with PacBio data. First, we supplied the 30 GB of raw PacBio reads to two different long read assemblers: Canu v1.7 (Koren et al. 2017) and to wtdbg2 v2.2 (Ruan, J, and Li H. unpublished data). Canu error-corrects raw reads prior to assembly whereas wtdbg2 assembles raw reads and then corrects the assembly based on consensus. Canu was run with an error correction rate of 8.5% (correctedErrorRate = 0.085). The corrected and trimmed reads used in the Canu assembly represented 16× coverage of the genome. For the wtdbg2 assembly, reads shorter than 5,000 bp in length were discarded (-L 5000), resulting in 22× coverage of the genome. Each of the two PacBio-only assemblies were polished with Pilon v1.21 (Walker et al. 2014) using 97× Illumina paired-end reads. Second, we used the PBSuite v15.8.24 (English et al. 2012) program PBJelly2 to conduct scaffolding and gap-filling of the Illumina assembly with the raw PacBio reads. The PBJelly2 assembly was then polished in Pilon with 97× Illumina paired-end reads.

We used two rounds of quickmerge (Alhakami et al. 2017) to merge the PBJelly, Canu, and wtdbg2 assemblies. First, we merged the Canu assembly with the wtdbg2 assembly using the Canu assembly as the reference. Second, we merged the PBjelly assembly with the Canu-wtdbg2 merged assembly using the Canu-wtdbg2 assembly as the reference. For both rounds of assembly merging, contigs were merged using a minimum alignment length of 7 kb (-ml 7000) and a 3.5 Mb length cutoff (-l 3500000) for anchor contigs. Lastly, we performed another round of polishing with Pilon, resulting in the final assembly.

### Transcriptome Sequencing

To assist in genome annotation, we sequenced and assembled a transcriptome for the orangethroat darter. We isolated RNA from one adult male and one adult female collected from the same location as the male used for genome sequencing. Additionally, we isolated RNA from a 1-week-old fry. The adults and fry were euthanized using an overdose of buffered MS-222 and placed on ice. Tissue from the eye, gonads, muscle, liver, fins, and brain from each adult and the entire fry were isolated and homogenized with a mortar and pestle. A Qiagen RNeasy extraction kit was used to isolate RNA from each tissue sample. We pooled equal amounts of RNA from the fry and each of the adult tissues to obtain a total of 1 μg RNA.

An RNAseq library was prepared and sequenced at the Biotechnology Center at UIUC. A TruSeq Stranded mRNAseq Sample Prep kit (Illumina) was used to prepare the RNAseq library, but modified so that fragmentation was done at 80 °C for 2 min. Resulting cDNA fragments ranged from 100 to 900 bp, with an average of 400 bp. The RNAseq library was sequenced together with the 450 bp shotgun library (see above) to obtain paired-end 250 bp reads. The library was quantitated by qPCR. Sequencing was done on two lanes for 266 cycles from each end of the fragments on a HiSeq2500 machine using a HiSeq Rapid SBS sequencing kit (version 2). This resulted in a total of 128,023,978 reads (64,011,989 forward and 64,011,989 reverse reads).

### Transcriptome Assembly

We used the program *process_shortreads* in Stacks to remove adaptors and poor-quality reads present in the RNAseq library. After quality filtering, 127,489,576 paired reads were retained and used to create a de novo transcriptome assembly with Trinity v2.5.1 (Grabherr et al. 2011). To determine the percent of the RNAseq reads that were represented in the Trinity assembly, we used Bowtie2 (v2.3.3.1) (Langmead and Salzberg 2012) to align the RNAseq reads back to the assembled transcripts. We also evaluated the number of Actinopterygii-specific BUSCOs present in the transcriptome assembly.

### Genome Annotation

We executed three iterative rounds of the Maker v2.31.9 (Cantarel et al. 2007) genome annotation pipeline to predict protein-coding genes. We supplied the following evidence to Maker for the first round of annotation: the orangethroat darter genome assembly, the orangethroat darter transcriptome, protein sequences obtained from five other teleost species (large yellow croaker *Larimichthys crocea* NCBI ASM74293v1, threespine stickleback *Gasterosteus aculeatus* Ensembl BROAD S1, zebrafish *Danio rerio* Ensembl GRCz11, medaka *Oryzias latipes* Ensembl HdrR, and tilapia *Oreochromis niloticus* Ensembl Orenil1.0), and the entire set of UniProt Swiss-Prot proteins (http://www.uniprit.org/; last accessed November 4, 2019) (Small et al. 2016). Maker used a list of known transposable elements and a RepBase library to soft mask repetitive elements prior to the initial annotation. We also provided Maker with an orangethroat darter-specific repeat library that was generated with RepeatModeler (v1.0.11; Smit and Hubley 2008).

We used the gene predictions produced by the first round of annotation in Maker to train SNAP and Augustus for use in subsequent rounds of gene prediction. When training SNAP, we included gene models with a maximum annotation edit distance (AED) of 0.25 and a minimum length of 50 amino acids. We then conducted the second round of Maker with ab initio gene prediction by supplying the transcript, protein, and repeat alignments generated in the first round of annotation, and enabling gene prediction by SNAP and Augustus using the models produced from training. After completion of the second round of Maker, we used the resulting transcripts to retrain SNAP and Augustus and then performed a third round of Maker with ab initio gene predictions. We analyzed the quality of the transcripts produced by the third round of Maker with BUSCO, using the orangethroat darter-specific Augustus model. To conduct functional annotation of proteins, we used BlastP (NCBI) to identify putative matches between the orangethroat darter proteins and those present in the UniProt Swiss-Prot database.

### Integrating the Orangethroat Linkage Map and Genome Scaffolds

We used Chromonomer v1.08 (http://http://catchenlab.life.illinois.edu/chromonomer/; last accessed November 4, 2019) to join and orient scaffolds from the orangethroat darter genome assembly into chromosomes. We aligned the 1,111 linkage map markers (i.e., 100 bp RAD tags) to the assembled orangethroat darter scaffolds with GSNAP (Wu et al. 2016). The resulting SAM file was provided to Chromonomer, along with AGP and FASTA files for the assembly and a file with the marker names and locations (in cM) for each of the 24 linkage groups.

### Synteny and Homology Analyses

To test for the presence of chromosomal rearrangements that putatively contribute to postzygotic isolation, we investigated synteny and homology between the rainbow darter linkage map and the orangethroat darter genome assembly. To infer which rearrangements might be unique to orangethroat versus rainbow darters, we also examined synteny between both darters and the closest relative for which a genome assembly is available, the yellow perch (*Perca flavescens*). Darters and perch are both in the family Percidae and are estimated to have last shared a common ancestor 58–66 Ma (Stepien and Haponski 2015). We downloaded the yellow perch genome assembly (PFLA_1.0) from NCBI’s GenBank.

We used Synolog (Small et al. 2016) to visualize pairwise alignments between the rainbow darter linkage map markers, the orangethroat darter genome assembly, and the yellow perch genome assembly. To scale the size of the rainbow darter linkage groups (in cM) for use in comparisons with the orangethroat darter and yellow perch genome assemblies (in Mb), we multiplied the position of each linkage marker in cM by 1,000,000 (equivalent to 1 Mb). Rainbow darter linkage map markers were mapped to the orangethroat darter and yellow perch assemblies in Synolog. The locations of the rainbow darter linkage map markers were then compared between the orangethroat darter and yellow perch genome assemblies in Synolog. Thus, the same set of markers was used in all three pairwise comparisons.

### Identifying Genetic Incompatibilities Using Backcross Genomes

Genomic regions associated with postzygotic barriers can be identified by quantifying patterns of introgression in hybrid genomes. Theory predicts that hybrid genomes will be more likely to exhibit nonadmixed haplotypes at areas of the genome associated with genetic incompatibilities, which can include both chromosomal rearrangements and negative epistatic interactions (Barton and Hewitt 1985; Barton and Bengtsson 1986; Rieseberg et al. 1999; Turelli et al. 2001). To identify genomic regions potentially underlying postzygotic isolation between orangethroat and rainbow darters, we examined patterns of local ancestry and deviations from Mendelian segregation (also called transmission ratio distortion) in backcross hybrid genomes. We then asked whether regions of the genome showing evidence of transmission ratio distortion overlap with: 1) regions of high genetic divergence (*F*_ST_, *D*_XY_) between parental species, and/or 2) regions showing evidence of chromosomal rearrangements between parental species.

We previously measured backcross viability in the laboratory by crossing wild-caught F1 hybrid males to parental females of both species and comparing their survival to parental control crosses (Moran et al. 2018). To generate the experimental backcrosses, six wild-caught F1 hybrid males were used in two cross-types (fig. 1*B*). Each F1 hybrid male was crossed with a female rainbow darter and with a female orangethroat darter. Backcross clutches suffered from significantly higher mortality rates compared with both parental and F1 hybrid clutches. On an average, only 7% of fry per backcross clutch survived at least 1-week posthatching, compared with 65% in parental crosses within each species (Moran et al. 2018). Backcrosses to orangethroat darters resulted in a total of 36 fry from two families that survived to 1-week posthatching. Backcrosses to rainbow darters resulted in a total of 13 fry from three families that survived to 1-week posthatching (see Moran et al. 2018 for details). Notably, one F1 hybrid male sired 35 of the surviving orangethroat-backcross fry and 10 of the surviving rainbow-backcross fry.

We generated and sequenced RAD data for the 49 total backcross offspring following the methods outlined above (see Linkage Map Sequencing—Orangethroat darter). We used the Stacks (v2.0; Rochette et al. 2019) program *process_radtags* to demultiplex the raw sequences resulting from the linkage map RADseq libraries (116,867,198 orangethroat-backcross raw reads; 27,581,894 rainbow-backcross raw reads), and to remove barcodes and low-quality reads. After quality filtering, we retained 116,424,777 orangethroat-backcross reads and 27,380,460 rainbow-backcross reads. To quantify patterns of genomic divergence between parental species and to facilitate identification of introgressed genomic regions in backcross offspring, we also obtained previously published RADseq data for 18 orangethroat and 18 rainbow darter individuals from NCBI’s Sequence Read Archive (SRP152572) (Moran et al. 2018). For our analyses, we only used individuals with <20% missing data and an ancestry fraction (Q) of >0.95 (see ADMIXTURE analysis below), indicating nonintrogressed individuals. This filtering resulted in a total of 10 orangethroat darters (5 females and 5 males) and 14 rainbow darters (9 females and 5 males). Reads from backcross fry and adult orangethroat and rainbow darters were aligned to the orangethroat darter genome with GSNAP and then supplied to the Stacks *ref_map* pipeline for RAD locus catalog construction and genotyping. The *ref_map* pipeline built and genotyped a total of 81,615 loci (i.e., 100 bp RAD tags) containing 117,524 SNPs with a mean ± SE coverage of 73.9× ±13.95 per individual across all loci.

We used the *populations* program in Stacks to further filter RAD loci for quality. We specified that a given RAD locus was only to be retained if present in all four populations (i.e., both sets of backcrosses and both parental species) and in a minimum of 50% of the individuals within each population. To filter out rare loci potentially originating from sequencing errors, we also excluded loci with a minor allele frequency (MAF) across all populations of <0.05. This resulted in a set of 29,064 SNPs across 19,772 loci (1.98 million sites) that were shared across both sets of backcross fry and both parental species.

We used the software ADMIXTURE (v1.3.0) (Alexander et al. 2009) to infer genome-wide ancestry proportions from each individual. Because ADMIXTURE assumes independence among SNPs, we kept only the first SNP in each RAD locus (resulting in 19,772 SNPs) for this analysis. We ran ADMIXTURE with 10,000 rounds of bootstrap resampling and specified two ancestral populations.

#### Mapping Local Ancestry across Backcross Hybrid Genomes

We used the total set of 29,064 shared SNPs obtained from Stacks to infer local ancestry along backcross hybrid linkage groups with ELAI (Efficient Local Ancestry Inference) v1.00 (Guan 2014). ELAI uses unphased genotype data to train and implement a two-level Hidden Markov model (HMM) to identify introgressed tracts in the genome. We trained the HMM using the nonadmixed individuals from both parental species and then predicted allele dosage along each linkage group for each backcross hybrid individual. We used a mean ± SE of 1,108 ± 56.17 SNPs per linkage group (1 SNP/27 kb) in model training and hybrid ancestry predictions. We expect that this density of SNPs should be sufficient to detect the majority of ancestry switches (i.e., junctions) across the genome for two reasons. First, we used second generation hybrids, which are predicted to have relatively large admixed haplotype blocks, as opposed to the smaller haplotype blocks that are typically observed in more advanced generations of hybrids. Our results are in agreement with this prediction.

Second, analyses of LD decay indicate that most ancestry blocks likely include multiple SNPs. We used genotype data from the ten nonintrogressed, wild-caught orangethroat darter individuals to estimate the average rate of LD decay between the set of 29,064 SNPs across the orangethroat darter genome in PopLDdecay (Zhang et al. 2018). Linkage between sites was measured as the squared Pearson coefficient of correlation, *r*^2^, which ranges from 0 to 1. Two sites are said to be in complete LD if they are tightly linked (i.e., not broken up by recombination), indicated by an *r*^2^ value of 1. Conversely, an *r*^2^ value of 0 indicates complete linkage equilibrium (i.e., no association) between sites. The average genome-wide linkage between physically linked sites (i.e., SNPs occurring on the same linkage group) decayed to *r*^2^ <0.5 by 100 kb and decayed to the mean background level of *r*^2^ <0.25 by 700 kb (supplementary fig. 1, Supplementary Material online). Thus, we expect that most ancestry blocks should be covered by multiple SNPs since we predict an average density of 1 SNP per 27 kb.

Mendelian segregation with at least one crossover event per chromosome predicts that, for a given chromosome, backcross offspring will be 50% recombinant, 25% nonrecombinant homozygotes (i.e., inherit two copies of a given chromosome from the same parental species), and 25% nonrecombinant heterozygotes (i.e., inherit one chromosome from each parental species) (fig. 1*B*). For each backcross individual, we scored each linkage group as recombinant, nonrecombinant homozygous, or nonrecombinant heterozygous and used χ^2^ tests to ask whether backcross offspring deviated from these expected frequencies across all 24 linkage groups. We then used binomial tests to ask whether individual linkage groups deviated from the expected frequencies. All statistical analyses were conducted in R (v3.4.4).

#### Deviations from Mendelian Segregation in Backcrosses

Genomic loci that deviate from the expected Mendelian segregation ratios in hybrids may contain genes associated with hybrid lethality (Hall and Willis 2005). Transmission ratio distortion in hybrids has been linked to chromosomal rearrangements and negative epistatic interactions (Rieseberg et al. 1995; De Villena and Sapienza 2001; Leppälä et al. 2013). To identify loci showing transmission ratio distortion in backcross hybrid genomes, we first filtered the set of 29,064 shared SNPs to include only SNPs that were differentially fixed between the parental species. This resulted in a set of 17,611 fixed SNPs across 8,662 loci. We further filtered this SNP set to only include those mapped to 1 of the 24 linkage groups, resulting in 16,585 SNPs across 8,177 loci. Because SNPs occurring together on the same 100-bp RAD tag are physically linked, we excluded all but the first SNP from each locus, resulting in 8,177 independent SNPs. Mendelian segregation predicts a MAF of 0.25 across all loci in a backcross population. We tested for a deviation from the expected MAF in both sets of backcross individuals (i.e., the 36 orangethroat-backcross fry and the 13 rainbow-backcross fry). We used Plink v1.9 (Purcell and Chang 2019) to calculate allele frequencies at each SNP in both backcross groups. To test for deviations from the expected MAF of 0.25 at each SNP, we performed χ^2^ tests with 1 degree of freedom. To control for multiple tests, we applied a 5% false discovery rate (FDR) using the Benjamini and Hochberg (1995) procedure in the p.adjust function of the R package *stats* (R Core Team 2013).

### Patterns of Recombination Rate Variation across Parental Genomes

Examining recombination rate across the genome can help identify certain aspects of genomic architecture. For example, centromeres are predicted to show a reduction in recombination rate relative to the background level whereas regions of markedly increased recombination rate may indicate structural polymorphisms within a population or a mis-assembly in the reference genome. Furthermore, theory predicts that loci important to reproductive isolation between hybridizing species may be more likely to occur in regions of low recombination (Bürger and Akerman 2011; Yeaman and Whitlock 2011; Flaxman et al. 2014). If recombination is reduced near adaptive alleles relative to the genomic background level, it can prevent the breakup of coadapted alleles and allow genetic differentiation to persist in the face of gene flow (Ortiz-Barrientos et al. 2016). Accordingly, we might expect regions of the genome associated with reproductive barriers to exhibit low recombination and high genetic differentiation between hybridizing species (see below). To estimate the population-level recombination rate, rho (*ρ = *4 *N*_e_*r*), across the genome, we provided the set of 29,064 shared SNPs identified above to the Interval program in LDhat v2.2 (McVean and Auton 2007). Calculations were conducted separately for both species using the 10 wild-caught orangethroat darters and 14 wild-caught rainbow darters with ancestry proportions >0.95 (see above). Interval uses a reversible-jump MCMC algorithm to estimate recombination rates from population data. We specified a burn-in of 100,000 iterations followed by 2,000,000 iterations with sampling every 5,000 iterations and a block penalty of 1. This allowed us to obtain an estimate of *ρ* across each linkage group for both species. As the population-level recombination rate assumes that individuals included in the analysis are unrelated, we were unable to calculate genome-wide estimates of *ρ* for the backcross offspring.

### Genetic Differentiation between Species

Regions of the genome showing elevated levels of genetic divergence between hybridizing species may indicate reproductive barriers that are resistant to gene flow (Noor and Bennett 2010; Nosil and Feder 2012). Conversely, regions with low genetic divergence can indicate regions that have high permeability to gene flow and/or regions that are identical by descent and have not been under strong divergent selection between species. Our goal was to determine whether regions of the genome exhibiting high levels of transmission ratio distortion in backcross hybrids and chromosomal rearrangements between species also show high levels of genetic divergence between species. We used the reference-aligned RADseq data from adult, wild-caught orangethroat darters (*n* = 10) and rainbow darters (*n* = 14) described earlier to calculate genome-wide population genomic statistics in Stacks. We used the Stacks v1.48 *populations* program to select loci that were present in at least 50% of the individuals within each population (i.e., orangethroat darters and rainbow darters). We also specified a MAF cutoff of 0.05. This resulted in a set of 43,502 SNPs across 16,950 RAD loci (∼1.6 million sites), with 39,518 SNPs mapping to 1 of the 24 linkage groups. To assess levels of genetic diversity across the genome within each species, we calculated the nucleotide diversity (π) at invariant and variant site using *populations*. We conducted genome scans for regions of elevated genetic differentiation between orangethroat and rainbow darters by calculating *F*_ST_ at each SNP and the smoothed amova*F*_ST_ in 500 kb sliding windows across each linkage group in *populations*. We also calculated absolute genetic divergence (*D*_XY_; Nei and Li 1979) in 50 kb nonoverlapping windows across all variant and invariant sites using a custom Python script. This resulted in 3,443 windows total and included 0.81 million sites across the genome. Windows with absolute genetic divergence falling above the 99th percentile were designated as *D*_XY_ outliers.

To test for a relationship between genetic divergence between species and transmission ratio distortion in backcrosses, we performed Pearson correlations between *D*_XY_ and the average absolute deviation from the expected MAF in backcrosses in 50 kb windows across the genome. To ask whether regions of high genetic divergence are more likely to occur in regions of reduced recombination, we also performed correlations between the population-level recombination rate, *ρ*, in both species and *D*_XY_ in 50 kb windows across the genome, in R.

### Gene Ontology Enrichment Analysis

We used the orangethroat darter genome annotation to identify genes present within each 50 kb *D*_XY_ outlier window, in addition to any genes within 20 kb of an outlier. To identify any overrepresented biological processes associated with the genes found in outlier regions, we used the Gene Ontology (GO) Consortium resources (Ashburner et al. 2000) to perform a GO enrichment analysis. We supplied the GO Enrichment Analysis tool with zebrafish (*Danio rerio*) orthologs for the genes we identified as outliers in the orangethroat darter genome annotation and specified the zebrafish database as the reference list. Overrepresented biological processes were identified using a Fisher’s exact test (*P* < 0.05) in PANTHER (v14.1) (Huaiyu et al. 2019). We used REVIGO (Supek et al. 2011) to perform a semantic similarity analysis with a similarity cutoff of 0.5 and the SimRel measure of semantic similarity, which allowed us to cluster and visualize GO terms by relatedness. 

## Supplementary Material

msz260_Supplementary_Data
