# Supplementary material for: Genomic Resources for Darters (Percidae: Etheostominae) Provide Insight into Postzygotic Barriers Implicated in Speciation
Source: Mol Biol Evol. 2019 Nov 5;37(3):711–29. doi: 10.1093/molbev/msz260 (PMC7038671; doi:10.1093/molbev/msz260)
Supplement: msz260_Supplementary_Data [file msz260_supplementary_data.zip › SupplementaryMaterial_MBE.pdf]

SUPPLEMENTARY TABLES

Supplementary Table 1. Statistics for the Meraculous genome assemblies using various kmer lengths produced with Illumina shotgun and mate-pair libraries.

kmer length	# scaffolds 1kb+	scaffold sequence total (Mb)	# contigs	% gaps in assembly	scaffold N50 (Mb)	contig N50 (kb)	# scaffolds > 50 kb	% genome in scaffolds > 50 kb
49	4623	717.5	368,313	11.6	2.4	6.2	805	95.7
59	4629	719.8	277,301	10.7	2.2	7.8	839	96.1
69	4988	716.4	220,375	10.1	2.5	9.3	781	95.8
79	5429	714.3	193,878	9.5	2.3	10.1	773	95.6

Supplementary Table 2. The number of Actinopterygii-specific Benchmarking Universal Single-Copy Orthologs (BUSCOs) present in each of the Meraculous genome assemblies produced using Illumina short-read sequencing.

kmer length	Complete BUSCOs	Complete single-copy BUSCOs	Complete and duplicated BUSCOs	Fragmented BUSCOs	Missing BUSCOs	Total BUSCO groups searched
49	4347 (94.8%)	4247 (92.6%)	100 (2.2%)	86 (1.9%)	151 (3.3%)	4584
59	4334 (94.5%)	4241 (92.5%)	93 (2.0%)	93 (1.9%)	157 (3.5%)	4584
69	4331 (94.4%)	4233 (92.3%)	98 (2.1%)	86 (1.9%)	167 (3.7%)	4584
79	4320 (94.2%)	4219 (92.0%)	101 (2.2%)	91 (2.0%)	173 (3.8%)	4584

Supplementary Table 3. Total amount of sequence data retained for each Illumina library after quality filtering. Coverage calculation is based on an estimated genome size of 1 Gb.

Library	Read length	# Reads retained	# Total bps	Coverage
450 bp shotgun	250	388,991,066	97,247,766,500	97X
800 bp shotgun	150	63,200,545	10,112,087,200	10X
3-5 kb mate-pair	150	79,139,172	12,662,267,520	13X
5-7 kb mate-pair	150	76,273,856	12,203,816,960	12X
8-12 kb mate-pair	150	78,571,066	12,571,370,560	13X

Supplementary Table 4. The number of Actinopterygii-specific Benchmarking Universal Single-Copy Orthologs (BUSCOs) present in the transcriptome assembly.

Complete BUSCOs	Complete single-copy BUSCOs	Complete and duplicated BUSCOs	Fragmented BUSCOs	Missing BUSCOs	Total BUSCO groups searched
4370 (95.3%)	4370 (31.0%)	2949 (64.3%)	150 (3.3%)	64 (1.4%)	4584

Supplementary Table 5. Proportion of homozygous, recombinant, and heterozygous haplotypes observed at each linkage group (LG) in backcrosses to orangethroat darters and P values from binomial tests for deviations from the expected proportions (i.e., 0.25 homozygous, 0.50 recombinant, and 0.25 heterozygous for each linkage group). Significant P values (<0.05) are bolded. CI= Confidence interval, Prop = proportion. *Linkage groups involved in translocations.

	**Homozygous**				**Recombinant**				**Heterozygous**			
**LG**	**Prop**	**CI low**	**CI high**	**P value**	**Prop**	**CI low**	**CI high**	**P value**	**Prop**	**CI low**	**CI high**	**P value**
1*	0.86	0.71	0.95	**<0.0001**	0.11	0.03	0.26	**<0.0001**	0.03	0	0.15	**<0.001**
2	0.81	0.64	0.92	**<0.0001**	0.17	0.06	0.33	**<0.001**	0.03	0	0.15	**<0.001**
3*	0.94	0.81	0.99	**<0.0001**	0.06	0.01	0.19	**<0.0001**	0	0	0.1	**<0.001**
4*	0.75	0.58	0.88	**<0.0001**	0.25	0.12	0.42	**<0.01**	0	0	0.1	**<0.001**
5*	0.42	0.26	0.59	**0.03**	0.58	0.41	0.74	0.41	0	0	0.1	**<0.001**
6	0.83	0.67	0.94	**<0.0001**	0.14	0.05	0.29	**<0.0001**	0.03	0	0.15	**<0.001**
7	0.83	0.67	0.94	**<0.0001**	0.14	0.05	0.29	**<0.0001**	0.03	0	0.15	**<0.001**
8*	0.69	0.52	0.84	**<0.0001**	0.28	0.14	0.45	**0.011**	0.03	0	0.15	**<0.001**
9	0.75	0.58	0.88	**<0.0001**	0.19	0.08	0.36	**<0.001**	0.06	0.01	0.19	**<0.01**
10	0.67	0.49	0.81	**<0.0001**	0.33	0.19	0.51	0.07	0	0	0.1	**<0.001**
11*	0.83	0.67	0.94	**<0.0001**	0.17	0.06	0.33	**<0.001**	0	0	0.1	**<0.001**
12	0.75	0.58	0.88	**<0.0001**	0.25	0.12	0.42	**<0.01**	0	0	0.1	**<0.001**
13	0.61	0.43	0.77	**<0.0001**	0.33	0.19	0.51	0.07	0.06	0.01	0.19	**<0.01**
14*	0.64	0.46	0.79	**<0.0001**	0.31	0.16	0.48	**0.03**	0.06	0.01	0.19	**<0.01**
15*	0.64	0.46	0.79	**<0.0001**	0.36	0.21	0.54	0.13	0	0	0.1	**<0.001**
16*	0.81	0.64	0.92	**<0.0001**	0.19	0.08	0.36	**<0.001**	0	0	0.1	**<0.001**
17*	0.83	0.67	0.94	**<0.0001**	0.14	0.05	0.29	**<0.0001**	0.03	0	0.15	**<0.001**
18	0.81	0.64	0.92	**<0.0001**	0.19	0.08	0.36	**<0.0001**	0	0	0.1	**<0.001**
19	0.75	0.58	0.88	**<0.0001**	0.19	0.08	0.36	**<0.001**	0.06	0.01	0.19	**<0.01**
20	0.42	0.26	0.59	**0.03**	0.47	0.3	0.65	0.87	0.11	0.03	0.26	0.06
21	0.86	0.71	0.95	**<0.0001**	0.11	0.03	0.26	**<0.0001**	0.03	0	0.15	**<0.001**

22	0.81	0.64	0.92	**<0.0001**	0.19	0.08	0.36	**<0.0001**	0	0	0.1	**<0.001**
23	0.72	0.55	0.86	**<0.0001**	0.17	0.06	0.33	**<0.001**	0.11	0.03	0.26	0.06
24	0.92	0.78	0.98	**<0.0001**	0.06	0.01	0.19	**<0.0001**	0.03	0	0.15	**<0.001**

Supplementary Table 6. Proportion of homozygous, recombinant, and heterozygous haplotypes observed at each linkage group (LG) in backcrosses to rainbow darters and P values from binomial tests for deviations from the expected proportions (i.e., 0.25 homozygous, 0.50 recombinant, and 0.25 heterozygous for each linkage group). Significant P values (<0.05) are bolded. CI= Confidence interval, Prop = proportion. *Linkage groups involved in translocations.

	**Homozygous**				**Recombinant**				**Heterozygous**			
**LG**	**Prop**	**CI low**	**CI high**	**P value**	**Prop**	**CI low**	**CI high**	**P value**	**Prop**	**CI low**	**CI high**	**P value**
1*	0.62	0.32	0.86	**<0.01**	0.23	0.05	0.54	0.09	0.15	0.02	0.45	0.54
2	0.46	0.19	0.75	0.10	0.46	0.19	0.75	1.00	0.08	0.00	0.36	0.21
3*	0.69	0.39	0.91	**<0.01**	0.31	0.09	0.61	0.27	0.00	0.00	0.25	**0.048**
4*	0.38	0.14	0.68	0.33	0.38	0.14	0.68	0.58	0.23	0.05	0.54	1.00
5*	0.38	0.14	0.68	0.33	0.62	0.32	0.86	0.58	0.00	0.00	0.25	**0.048**
6	0.46	0.19	0.75	0.10	0.31	0.09	0.61	0.27	0.23	0.05	0.54	1.00
7	0.31	0.09	0.61	0.75	0.46	0.19	0.75	1.00	0.23	0.05	0.54	1.00
8*	0.38	0.14	0.68	0.33	0.62	0.32	0.86	0.58	0.00	0.00	0.25	**0.048**
9	0.69	0.39	0.91	**<0.01**	0.23	0.05	0.54	0.09	0.08	0.00	0.36	0.21
10	0.31	0.09	0.61	0.75	0.69	0.39	0.91	0.27	0.00	0.00	0.25	**0.048**
11*	0.38	0.14	0.68	0.33	0.31	0.09	0.61	0.27	0.31	0.09	0.61	0.75
12	0.31	0.09	0.61	0.75	0.38	0.14	0.68	0.58	0.31	0.09	0.61	0.75
13	0.54	0.25	0.81	**0.02**	0.23	0.05	0.54	0.09	0.23	0.05	0.54	1.00
14*	0.38	0.14	0.68	0.33	0.38	0.14	0.68	0.58	0.23	0.05	0.54	1.00
15*	0.54	0.25	0.81	**0.02**	0.38	0.14	0.68	0.58	0.08	0.00	0.36	0.21
16*	0.46	0.19	0.75	0.10	0.46	0.19	0.75	1.00	0.08	0.00	0.36	0.21
17*	0.77	0.46	0.95	**<0.0001**	0.23	0.05	0.54	0.09	0.00	0.00	0.25	**0.048**
18	0.46	0.19	0.75	0.10	0.46	0.19	0.75	1.00	0.08	0.00	0.36	0.21
19	0.54	0.25	0.81	**0.02**	0.15	0.02	0.45	**0.02**	0.31	0.09	0.61	0.75
20	0.23	0.05	0.54	1.00	0.77	0.46	0.95	0.09	0.00	0.00	0.25	**0.048**
21	0.69	0.39	0.91	**<0.01**	0.15	0.02	0.45	**0.02**	0.15	0.02	0.45	0.54

22	0.38	0.14	0.68	0.33	0.46	0.19	0.75	1.00	0.15	0.02	0.45	0.54
23	0.69	0.39	0.91	**<0.01**	0.00	0.00	0.25	**<0.001**	0.31	0.09	0.61	0.75
24	0.54	0.25	0.81	**0.02**	0.15	0.02	0.45	**0.02**	0.31	0.09	0.61	0.75

See Excel File for Supplementary Tables 7-9.

SUPPLEMENTARY FIGURES

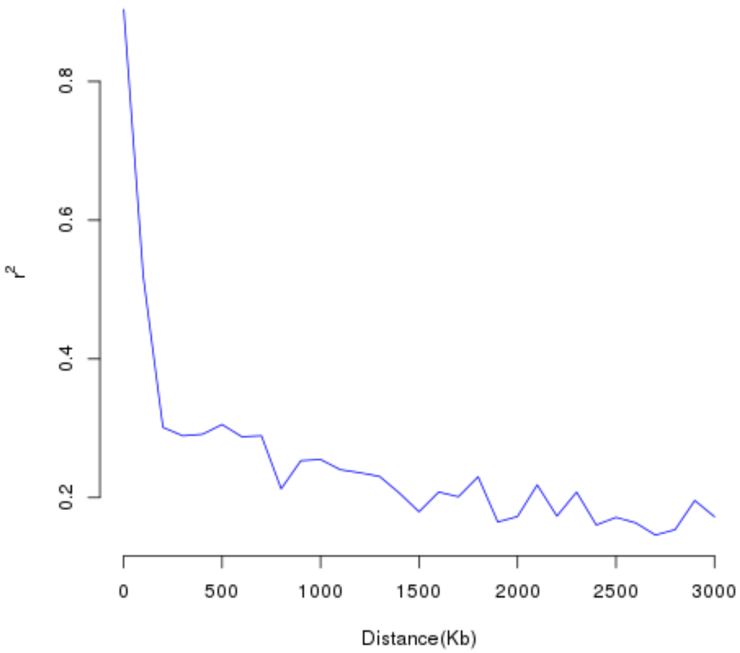

Supplementary Figure 1. Linkage disequilibrium decay (r^2) calculated between pairs of SNPs across the orangethroat darter genome.

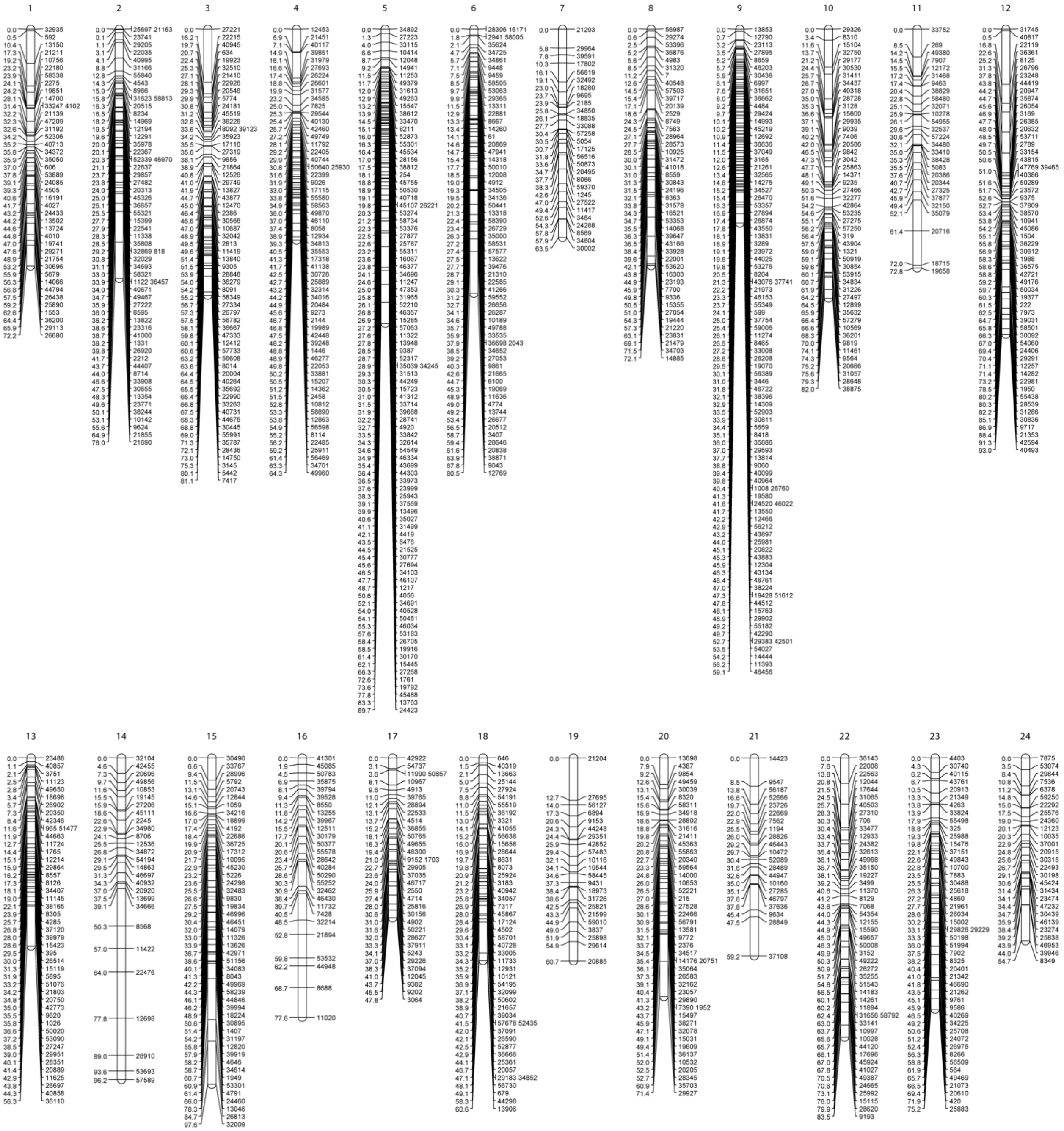

Supplementary Figure 2. Positions in cM for the 1,111 markers in the orangethroat darter consensus map.

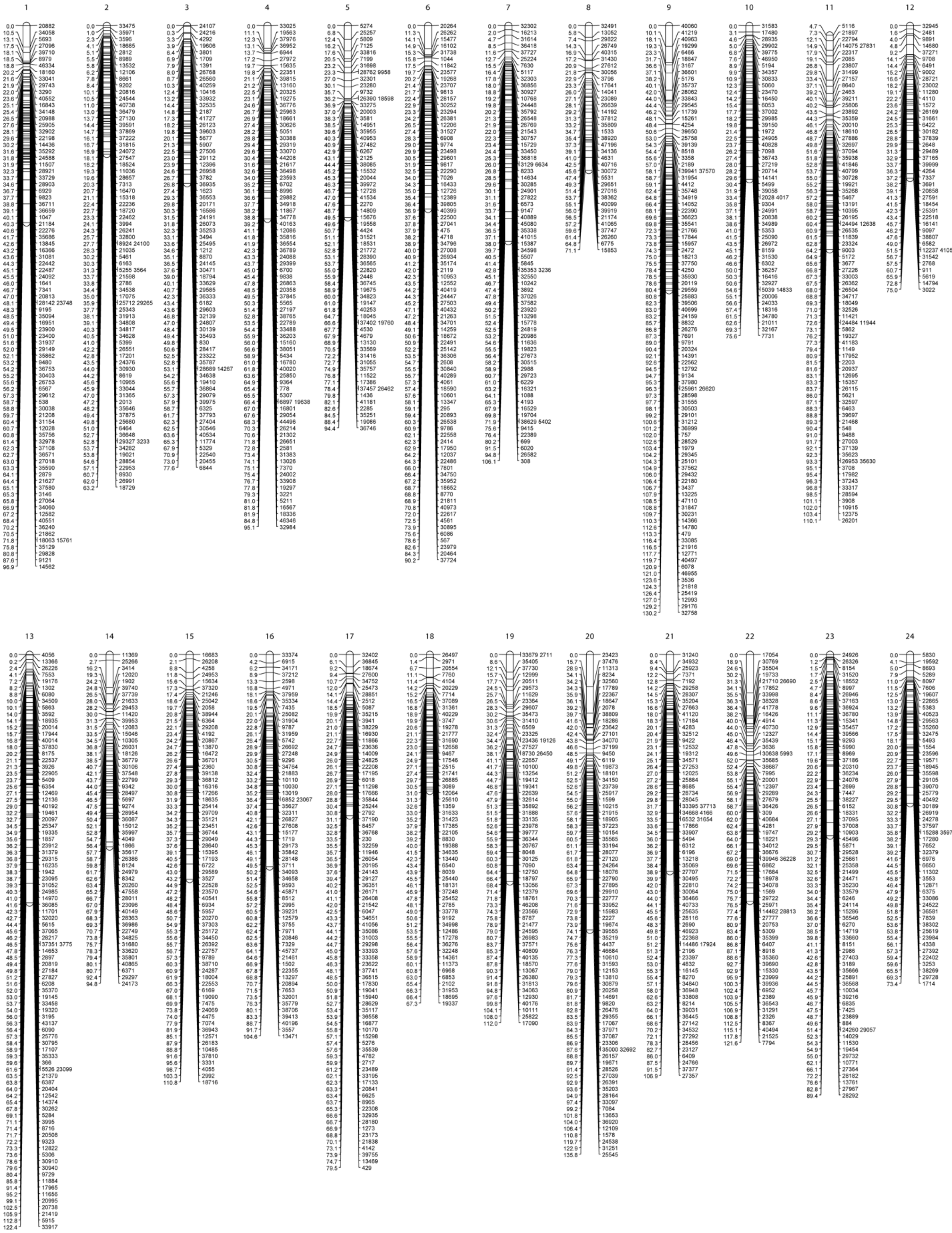

Supplementary Figure 3. Positions in cM for the 1,616 markers in the rainbow darter consensus map.

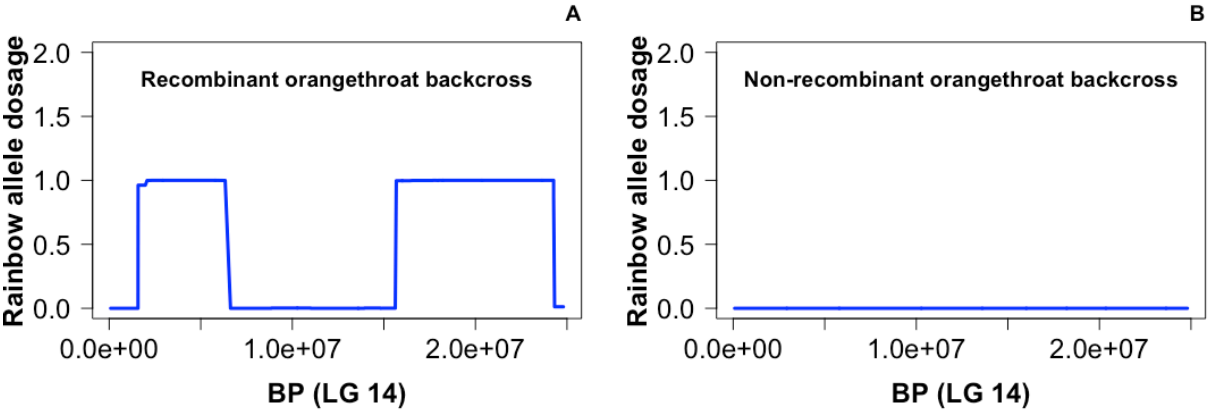

Supplementary Figure 4. Local ancestry along linkage group 14 for (A) a recombinant orangethroat-backcross individual and (B) a non-recombinant orangethroat-backcross individual. Both backcross individuals shown originated from the same family, which was produced by crossing an F1 hybrid male to an orangethroat female. The minor parent (i.e. rainbow darter, "RB") allele dosage is shown on the y axis. An allele dosage of 1 represents admixed regions of the genome with one rainbow allele and one orangethroat allele. An allele dosage of 0 represents non-admixed regions of the genome with zero rainbow darter alleles and two orangethroat darter alleles.

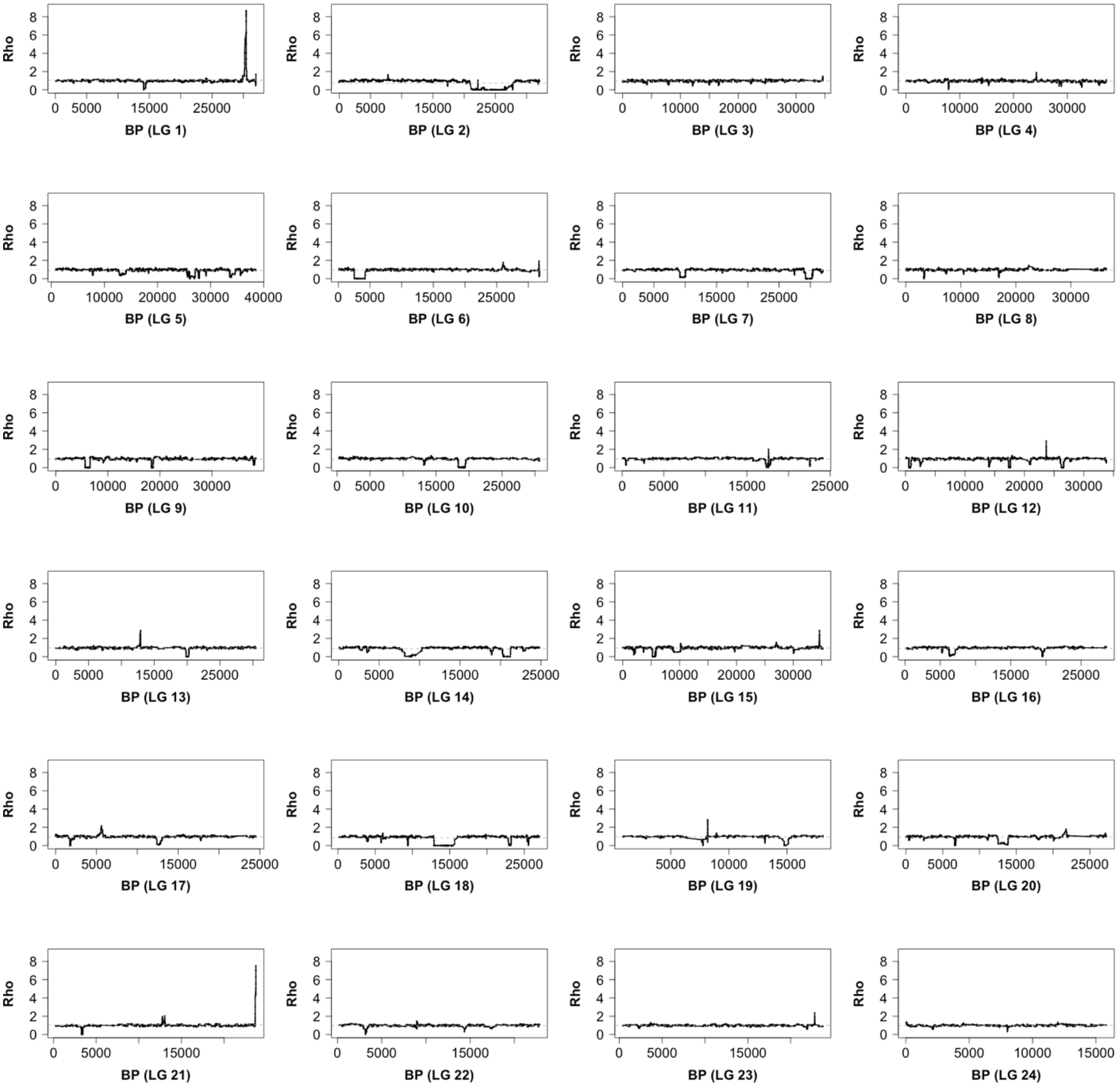

Supplementary Figure 5. Genome-wide population level estimates of the recombination rate, rho ($\rho = 4N_e r$), for orangethroat darters. Dashed gray lines represented the mean recombination rate for a given linkage group (LG).

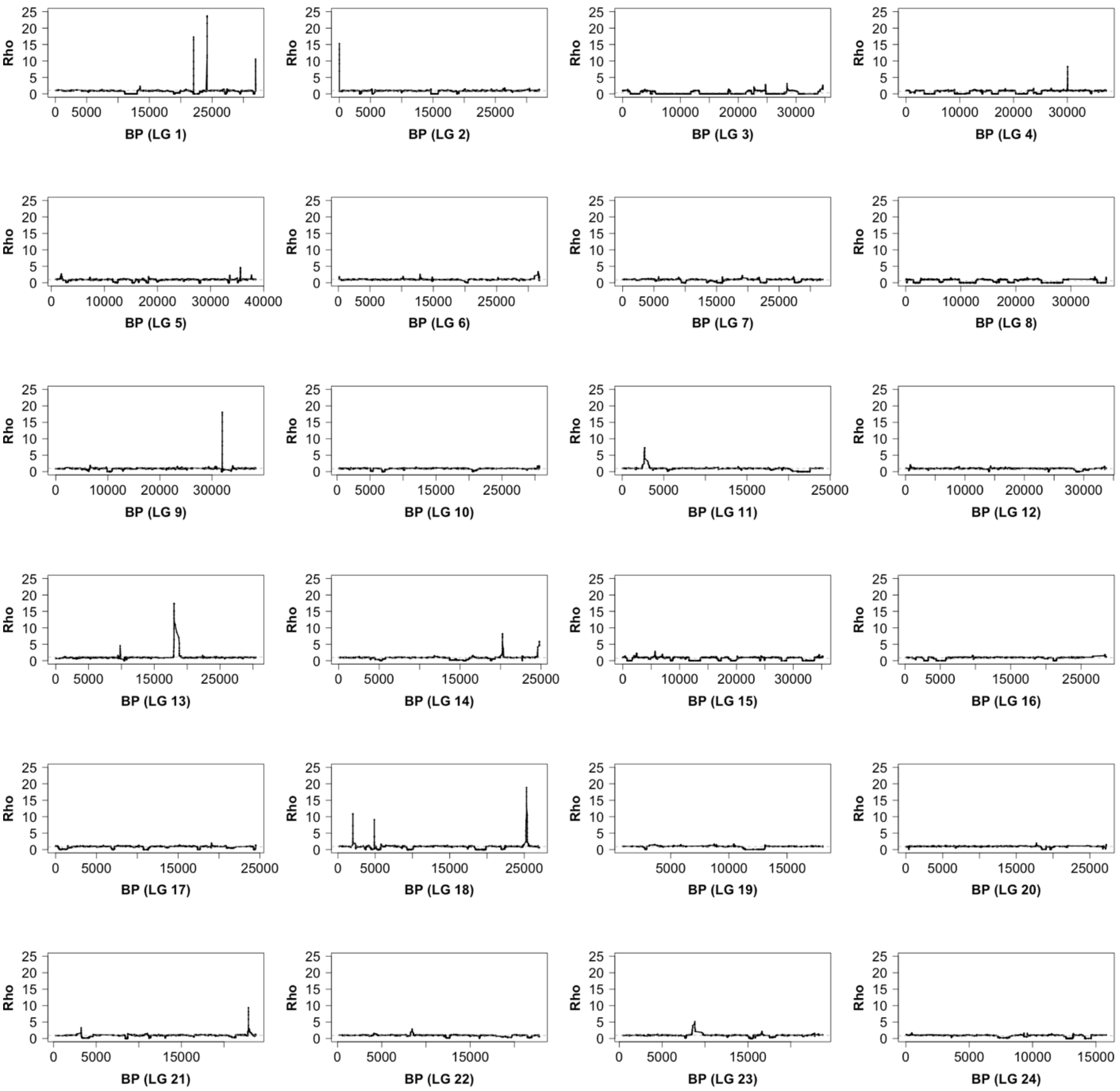

Supplementary Figure 6. Genome-wide population level estimates of the recombination rate, rho ($\rho = 4N_e r$), for rainbow darters. Dashed gray lines represented the mean recombination rate for a given linkage group (LG).

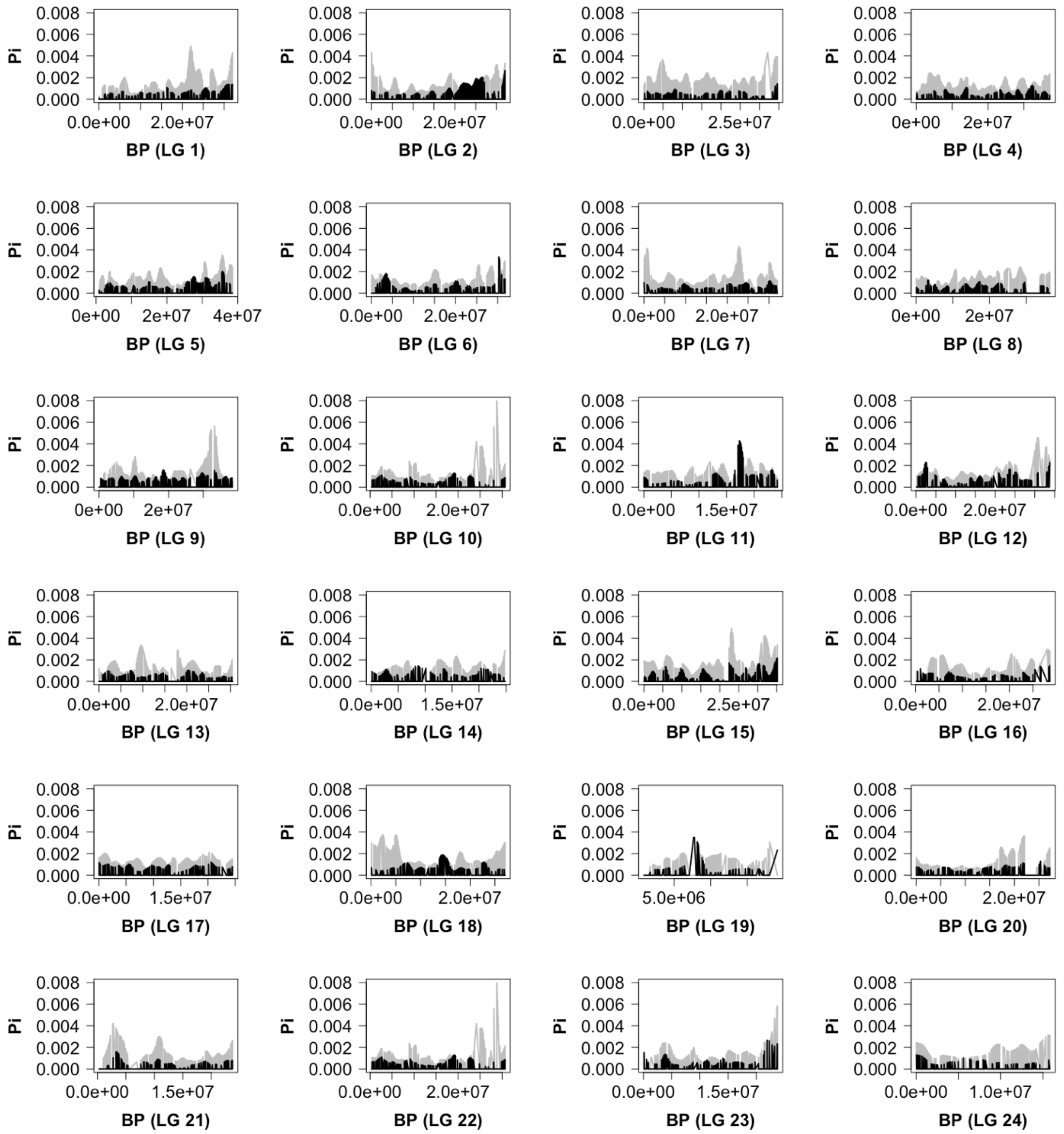

Supplementary Figure 7. Smoothed nucleotide diversity, π (Pi), within orangethroat darters (black) and rainbow darters (gray) for 44,688 sites across the 24 linkage groups (LG).
